# Thermo-responsive cascade antimicrobial platform for precise biofilm removal and enhanced wound healing

**DOI:** 10.1093/burnst/tkae038

**Published:** 2024-09-25

**Authors:** Ting Du, Jiangli Cao, Zhannuo Zhang, Zehui Xiao, Jingbo Jiao, Zhiyong Song, Xinjun Du, Shuo Wang

**Affiliations:** State Key Laboratory of Food Nutrition and Safety, College of Food Science and Engineering, Tianjin University of Science and Technology, No. 29, Thirteenth Street, Binhai New Area, Tianjin 300457, PR China; State Key Laboratory of Food Nutrition and Safety, College of Food Science and Engineering, Tianjin University of Science and Technology, No. 29, Thirteenth Street, Binhai New Area, Tianjin 300457, PR China; State Key Laboratory of Food Nutrition and Safety, College of Food Science and Engineering, Tianjin University of Science and Technology, No. 29, Thirteenth Street, Binhai New Area, Tianjin 300457, PR China; State Key Laboratory of Food Nutrition and Safety, College of Food Science and Engineering, Tianjin University of Science and Technology, No. 29, Thirteenth Street, Binhai New Area, Tianjin 300457, PR China; State Key Laboratory of Food Nutrition and Safety, College of Food Science and Engineering, Tianjin University of Science and Technology, No. 29, Thirteenth Street, Binhai New Area, Tianjin 300457, PR China; College of Science, Huazhong Agricultural University, No. 1, Shizishan Street, Hongshan District, Wuhan 430070, PR China; State Key Laboratory of Food Nutrition and Safety, College of Food Science and Engineering, Tianjin University of Science and Technology, No. 29, Thirteenth Street, Binhai New Area, Tianjin 300457, PR China; Tianjin Key Laboratory of Food Science and Health, School of Medicine, Nankai University, No. 38 Tongyan Road, Haihe Education Park, Jinnan District, Tianjin 300071, PR China

**Keywords:** HMAPH nanocomposites, Photothermal, Photodynamic, Fenton reaction, Synergistic antibacteria, Biofilm, Wound healing, Infection

## Abstract

**Background:**

Bacterial infection, tissue hypoxia and inflammatory response can hinder infected wound repair. This study aimed to develop a multifunctional specific therapeutic photo-activated release nanosystem [HMPB@MB@AuNPs@PMB@HA (HMAPH)] by loading photosensitizer methylene blue (MB) into hollow mesoporous Prussian blue nanostructures and modifying the surface with gold particles, polymyxin B (PMB) and hydrophilic hyaluronic acid.

**Methods:**

The HMAPH was characterized using transmission electron microscopy, UV–vis, Fourier-transform infrared spectroscopy, X-ray diffraction and X-ray photon spectroscopy. The photothermal performance, iron ion release and free radical generation of the HMAPH were measured under different conditions to investigate its thermo-responsive cascade reaction. The antibacterial ability of HMAPH was investigated using live/dead fluorescence tests. The morphology and membrane integrity of *Pseudomonas aeruginosa* (*P. aeruginosa*) were investigated using transmission electron microscopy. The anti-biofilm activity of HMAPH was evaluated using crystal violet and SYBR Green I staining. Finally, we established a mouse model of a skin wound infected by *P. aeruginosa* to confirm the *in vivo* effectiveness of HMAPH. We used immunofluorescent staining, hematoxylin–eosin staining, Masson staining and enzyme-linked immunosorbent assay to examine whether HMAPH promoted wound healing and reduced inflammatory damage.

**Results:**

In this study, hyaluronic acid was decomposed under the action of hyaluronidase. Also, the exposed nanomaterials specifically bound to the outer membrane of *P. aeruginosa* through PMB to increase the membrane sensitivity to photodynamic treatment. Under dual-light irradiation, a large amount of iron ions released by HMAPH underwent a Fenton reaction with H_2_O_2_ in bacteria to generate hydroxyl radicals (•OH), enabling direct killing of cells by hyperthermia. Additionally, the photodynamic activity of MB released by photo-induced activation led to the generation of reactive oxygen species, achieving synergistic and effective inhibition of *P. aeruginosa*. HMAPH also inhibited biofilm formation and downregulated the expression of virulence factors. *In vivo* experiments revealed that HMAPH accelerated the healing of *P. aeruginosa*-infected wounds by promoting angiogenesis and skin regeneration, inhibiting the inflammatory response and promoting M1 to M2 polarization.

**Conclusions:**

Our study proposed a strategy against bacteria and biofilms through a synergistic photothermal–photodynamic–Fenton reaction, opening up new prospects for combating biofilm-associated infections.

## Graphical Abstract

**Graphical Abstract:**
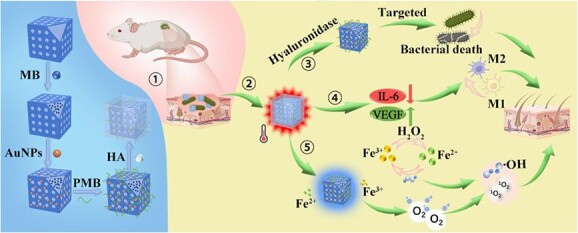

## Highlights

- A multifunctional specific therapeutic photo-activated release nanosystem (HMAPH) against biofilms and bacteria and promoting wound healing is developed.
- HMAPH can bind bacterial outer membrane through polymyxin B to increase bacterial membrane sensitivity to photodynamic treatment and provide favorable conditions for targeted and precise treatment of bacterial infections.
- Under dual NIR irradiation, a large amount of HMAPH-released iron ions reacted with H_2_O_2_ in bacteria to generate hydroxyl radicals (•OH), coupled with ROS generated by photodynamic activity of MB released by light-induced activation, achieving photothermal synergistic inhibition of bacterial infection.
- *In vivo* experiments revealed that HMAPH can accelerate *P. aeruginosa*-infected wound healing by promoting angiogenesis and skin regeneration, inhibiting the inflammatory response and promoting M1 to M2 polarization.

## Background

The rapid development of bacterial infections has become a major global health challenge, particularly in wound healing [1–3]. Open wounds are highly susceptible to infection by pathogenic bacteria, and the proliferation of pathogenic bacteria prolongs wound healing [4]. However, with antibiotic misuse, microbial infections are of significant concern, where the spread of drug-resistant microorganisms poses a significant challenge to public health [5–7]. *Pseudomonas aeruginosa (*P. aeruginosa*)* was recognized as a key priority pathogen by the World Health Organization in 2017 [8]. It shows natural resistance to antibiotics and stability in biofilm formation, thereby protecting bacteria from host immune defenses and antibiotics [9,10]. Self-produced extracellular polymeric substance renders bacteria in biofilms insensitive to antibiotics, thereby requiring a much higher effective dose than that against planktonic pathogens [11–13]. Additionally, with the emergence of an increasing number of multi-drug-resistant *P. aeruginosa* strains, studies have found that all clinically available anti-*Pseudomonas* antibiotics are becoming less effective in treatment [14]. Thus, new therapeutic methods need to be developed for the eradication of *P. aeruginosa* and its biofilm.

Recently, various emerging nanomaterial-based therapeutics have been widely used in antibacterial therapy, including chemotherapy [15,16], photothermal therapy (PTT) [17–19], photocatalytic therapy [20,21] and microwave therapy [22]. Among these, photodynamic therapy (PDT) and PTT have attracted increasing attention in antibacterial applications owing to their significant advantages, such as low invasion, fast onset of action and not-easy-to-develop drug resistance [23,24]. PDT is based on directly killing pathogens by cytotoxic reactive oxygen species (ROS) through destroying the bacterial cell membranes and DNA [25,26]. However, PTT kills bacteria by disrupting bacterial cell membranes and denaturing proteins through irradiating near-infrared (NIR) light at the target site and converting it into heat using a photothermal agent [23,27,28]. Presently, a single PDT or PTT cannot achieve a good therapeutic effect without damaging the surrounding tissues because of insufficient ROS produced, extremely high temperature or lack of selectivity. Conversely, the combination of PDT and PTT can enhance antimicrobial efficiency and reduce drug dose and laser power through their synergistic effect compared with the single-treatment mode [29–31]. Based on the pathogenic characteristics of *P. aeruginosa*, several challenges still need to be addressed before the clinical application of PDT and PTT. For instance, after biofilm formation, treatment using PDT and PTT is hindered by the presence of extracellular polymeric substance in mature thick biofilms, thus significantly limiting the diffusion depth of singlet oxygen and heat.

The catalytic conversion of hydrogen peroxide (H_2_O_2_) into hydroxyl radicals (•OH) based on *in situ* Fenton-like reactions is considered a promising strategy against bacterial infections [32]. Usually, high concentrations of H_2_O_2_ can kill bacteria, but they are harmful to normal tissues, suggesting the need to remove H_2_O_2_ during sterilization. Fe^2+^, Fe^3+^, Cu^+^, Mn^2+^ and other metal ions can be used to treat bacterial infections by catalyzing the conversion of H_2_O_2_ into •OH. Prussian blue (PB) is a typical mesoporous metal–organic framework (MOF) containing a large amount of Fe(II) and Fe(III) [33,34]. Also, iron ions can carry out the Fenton reaction by reacting with H_2_O_2_ in bacteria, leading to the formation of •OH with extremely strong oxidative capacity [35]. Hu *et al*. developed Cu_2_-xS nanodots for cancer therapy via synergistic nanocatalysis and PTT [36]. Xie *et al*. prepared copper peroxide-loaded tungsten disulfide nanoflowers by integrating the Fenton reaction and PTT for treating bacterial infections [37]. However, the Fenton reaction–PTT–PDT system is rarely reported in the field of antibacterial research. Thus, we hypothesized that the synergy of the Fenton reaction, PTT and PDT could effectively overcome the deficiencies of single-modal antimicrobial technologies and remove biofilms.

In this study, we constructed a therapeutic platform by combining the Fenton reaction, PDT and PTT for biofilm eradication *in vitro* and wound healing *in vivo*. We first synthesized hollow mesoporous PB nanoparticles (HMPB NPs) by controlled chemical etching. HMPB was selected because its huge internal hollow mesoporous structure could serve as a drug carrier, and the charge transfer of the Fe^3+^–N ≡ C–Fe^2+^ composition in the PB framework could impart to HMPB the properties of a photothermal agent. Then, methylene blue (MB), a photosensitizer generating singlet oxygen (^1^O_2_), was encapsulated in the inner cavity of HMPB to prepare HMPB@MB. Next, the photothermal properties of HMPB@MB were enhanced using the reduction–deposition method to grow gold (Au) NPs on its surface to synthesize HMPB@MB@AuNPs (HMA) nanocomposites. Subsequently, polymyxin B (PMB) was selected to functionalize HMA to form HMAP because PMB could bind to the outer membrane of gram-negative bacteria to improve their sensitivity to photodynamic effects. Finally, hyaluronic acid (HA) was added to synthesize HMAPH through coordination. HA was used as the outermost layer (a sealing layer) to prevent MB release, whereas the hyaluronidase (HAase) secreted by bacteria triggered the degradation of the HA layer, further accelerating the release of MB to achieve its antibacterial activity. *In vitro* experiments showed that 99.97% of *P. aeruginosa* could be killed within 10 min of dual-light irradiation (808 + 660 nm), and the biofilms could be effectively removed through the synergistic effect of PTT, PDT and Fenton reaction. Further *in vivo* results demonstrated the bactericidal activity of HMAPH in a mouse wound infection model. Overall, our proposed strategy could help in the precise treatment of bacterial infections without damaging normal tissues, suggesting its significant potential for clinical application.

## Methods

### Synthesis of PB, HMPB and HMPB@MB NPs

The synthesis of HMPB followed a previously reported study [19]. Briefly, polyvinyl pyrrolidone (PVP; 3.0 g) was dissolved in 40 ml of HCl (0.01 M) for complete dissolution by ultrasound, followed by the addition of 132 mg of K_3_[Fe(CN)_6_] and stirring for 30 min. After transferring the mixture to an oven at 80°C and incubation for 24 h, the pellets were collected by centrifugation (12 000 rpm, 10 min), followed by three washes with deionized water to obtain PB nanoparticles.

Next, PB (5.0 mg) and PVP (50 mg) were dissolved in 5.0 ml of HCl (1.0 M) and stirred for 3 h, followed by transferring the mixture to an autoclave and heating at 140°C for 4 h. Subsequently, the obtained mixture was centrifuged and washed three times with deionized water to collect the precipitate, followed by dispersion in an ethanol solution, stirring overnight, and two washes with deionized water to remove excess PVP on the surface and obtain the final product as HMPB NPs.

HMPB and MB were stirred overnight at room temperature in the dark at a mass ratio of 2 : 1, followed by centrifugation and three washes with deionized water to prepare HMPB@MB NPs.

### Synthesis of HMA nanocomposites

HMPB@MB (4.5 mg) was dissolved in deionized water and sonicated for 5 min, followed by the addition of HAuCl_4_ dropwise and reaction at 80°C for 30 min under agitation until the color turned dark green. Finally, after multiple centrifugations and washes with ultrapure water, the precipitate was obtained as HMA nanocomposite.

### Synthesis of HMPB@MB@AuNPs@PMB nanocomposites

Carboxyl-polyethylene glycol-thiol (COOH-PEG-SH) (10 mg) was dissolved in 1.0 ml of deionized water, followed by the addition of 1-(3-dimethylaminopropyl)-3-ethylcarbo diimide hydrochloride (50 μl, 50 mg/ml) and *N*-hydroxysuccinimide solution (50 μl, 25 mg/ml) and allowed to react for 1 h to activate the carboxyl groups. After adding HMA to the above solution, the reaction was continued for 8 h to form Au–S bonds, marked as solution A.

Subsequently, PMB (40 mg) was dissolved in 10 ml of NaCO_3_/NaHCO_3_ (pH 8.5), marked as solution B. After adding solution A (10 mg/ml, 200 μl) to solution B, the mixture was sonicated for 20 min and then stirred for 5 h at room temperature. Finally, the excess PMB was removed by centrifugal washing with aqueous HCl (pH = 5.0) and deionized water, and the resulting precipitates were collected as HMPB@MB@AuNPs@PMB (HMAP) nanocomposites.

### Synthesis of HMPB@MB@AuNPs@PMB@HA nanocomposites

HA (20 mg/ml) was mixed with HMPB@MB@AuNPs@PMB (2.0 mg/ml) and stirred for 4 h at room temperature in the dark. After centrifugation and washing with deionized water, the precipitates were obtained as HMPB@MB@AuNPs@PMB@HA (HMAPH) nanocomposites.

The methods for *in vitro* antibacterial experiments and animal experiments of the materials are shown in the experimental section of the supporting information (see online supplementary material).

### Statistical analysis

All data analysis was performed by GraphPad software and the results are presented as mean ± standard deviation (SD). The Student’s *t* test, one-way analysis of variance (ANOVA) and two-way ANOVA were used to analyze the difference between different treatment groups. Significant differences were considered at *p* < 0.05 and are expressed as ^*^p < 0.05, ^**^p < 0.01, ^***^p < 0.001 and ^****^p < 0.0001. The primer sequences used in this study are shown in Table S1.

## Results

### Characterization of HMAPH nanocomposite

**Figure 1:**
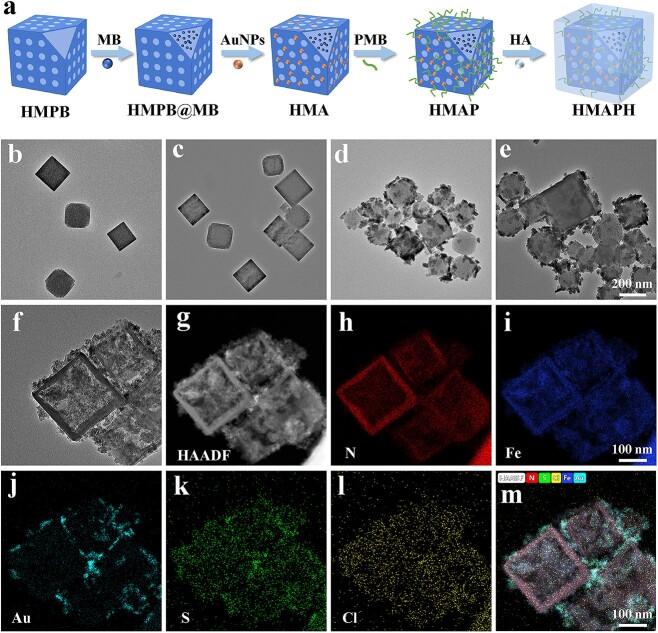
Characterization of HMAPH morphology. (**a**) Schematic diagram of the synthesis steps of HMAPH. TEM images of HMPB (**b**), HMPB@MB (**c**), HMA (**d**), HMAP (**e**) and HMAPH (**f**). (**g**–**m**) High-angle annular dark-field scanning TEM image of HMAPH and corresponding element mappings of N, Fe, Au, S and Cl. *HMA* HMPB@MB@AuNPs, *HMAP* HMPB@MB@AuNPs@PMB, *HMAPH* HMPB@MB@AuNPs@PMB@HA, *TEM* transmission electron microscopy, *PMB* polymyxin b, *HA* hyaluronic acid

**Figure 2:**
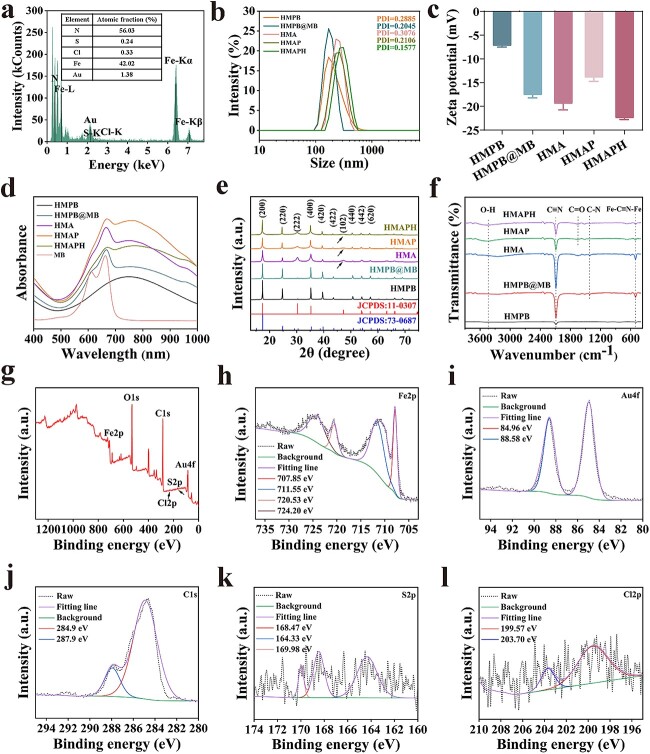
Characterization of HMAPH. (**a**) EDS analysis of HMAPH, with inset showing the content of each element. Hydrodynamic sizes (**b**) and zeta potential (**c**) of HMPB, HMPB@MB, HMA, HMAP and HMAPH. UV–vis spectra (**d**), XRD pattern (**e**) and FT-IR spectra (**f**) of HMPB, HMPB@MB, HMA, HMAP and HMAPH. XPS spectra of HMAPH (**g**) and high-resolution XPS spectra of Fe 2p (**h**), Au 4f (**i**), C 1 s (**j**), S 2p (**k**) and Cl 2p (**l**). *HMA* HMPB@MB@AuNPs, *HMAP* HMPB@MB@AuNPs@PMB, *HMAPH* HMPB@MB@AuNPs@PMB@HA, *a.u.* absorbance unit, *EDS* energy dispersive spectroscopy, *UV–vis* ultraviolet–visible spectroscopy, *XRD* X-ray diffraction, *FT-IR* fourier-transform infrared spectroscopy, *XPS* X-ray photoelectron spectroscopy, *PMB *polymyxin b

The process of designing and constructing HMAPH NPs is displayed in Figure 1a and Table S2 (see online supplementary material). HMPB was synthesized by a two-step method, with monodisperse PB NPs first formed by directional polymerization, followed by the preparation of hollow-structured HMPB through chemical etching with HCl at 140°C under the protection of PVP. Transmission electron microscopy (TEM) analysis revealed the morphology of HMPB as a typical cubic structure (Figure 1b). HMPB was better loaded with MB after rinsing with ethanol and water to remove PVP from its surface. In the TEM image of HMPB@MB in Figure 1c, the cubic structure of HMPB was not affected after MB loading. Figure 1d shows the preparation of HMA by reducing HAuCl_4_ to Au NPs on the HMPB@MB surface using an *in situ* growth method. The successful growth of Au *in situ* changed the solution color from blue to dark green. Also, the reaction of COOH–PEG–SH and HMA led to the formation of stable Au–S bonds, thereby modifying the Au surface with carboxyl groups. Next, PMB was attached to the surface via amide bonds under alkaline conditions to construct HMAP (Figure 1e). The functionalization of HMA by PMB to form HMAP could be ascribed to the destabilization of the bacterial outer membrane by the binding of PMB to the outer membrane of gram-negative bacteria, thereby enhancing the bacterial sensitivity to photodynamic treatment [38]. Finally, HA was coated on the outer surface to construct HMAPH so as to improve the hydrophilicity and biocompatibility of the material. Figure 1f displays the morphology of the prepared HMAPH. The successful preparation of HMAPH was also confirmed by high-angle annular dark-field scanning TEM elemental mapping and energy-dispersive spectroscopy. Figures 1g–m and 2a show the presence of Fe, N, Au, S and Cl elements, indicating the stepwise preparation of HMPB, HMPB@MB, HMA, HMAP and HMAPH.

As shown in Figure 2b, the hydrodynamic diameters showed narrow peaks for HMPB, HMPB@MB, HMA, HMAP and HMAPH NPs, with average values of 169.9, 169.9, 229.8, 267.2 and 310.7 nm, respectively. HMAPH showed a polydispersity index of 0.1577, suggesting good dispersibility (Figure 2b). Furthermore, we measured the hydrodynamic sizes of HMAPH in phosphate-buffered saline (PBS) over time. As shown in Figure S1a and b (see online supplementary material), HMAPH was highly stable in various physiological environments for a long time. Additionally, we evaluated the *in vitro* biodegradation behavior of HMAPH. Briefly, HMAPH (1.0 mg/ml) was immersed in PBS (30 ml) at different pH values (7.4, 6.5 and 6.0), which was used to simulate the slightly acidic microenvironment of neutral healthy body fluids and biofilms. As shown in Figure S1c, the solution color gradually faded from 0 h to 16 days with decreasing pH (from 7.4 to 6.0). Also, and the Fe(II)–CN–Fe(III) bond in HMAPH was unstable under mildly acidic conditions, allowing HMAPH to accelerate biodegradation behavior in specific weakly acidic microenvironments [39]. Consistent with the results in Figure S1c, the decline in the UV–vis absorbance of HMAPH at 725 nm was also pH dependent, with a much higher decline in a weakly acidic environment (pH 6.0) than in a neutral environment (pH 7.4) (Figure S1d). During the 16 days of the experiment, the characteristic absorption peaks of HMAPH at pH 6.0, 6.5 and 7.0 were 0.184, 0.274 and 0.665, respectively.

As shown in Figure 2c, the average zeta potential was −7.21 ± 0.31 mV for HMPB, which became more negative for HMPB@MB and HMA (−17.57 ± 0.65 mV and −19.40 ± 0.15 mV, respectively) due to MB loading and Au modification. The zeta potential became smaller (−13.90 ± 0.82 mV) after coating with PMB, and the zeta potential was further negatively charged (−22.40 ± 0.36 mV) upon HA modification. These changes in zeta potential indicated the successful modification of MB, Au particles and HA on the HMPB surface. Additionally, the drug-loading capacity and encapsulation efficiency of MB in HMAPH were calculated as 6.54 and 13.08%, respectively (Figure S2, see online supplementary material). As shown in Figure 2d, HMPB had a broad absorption peak from 650 to 850 nm in the UV–vis absorption spectrum, covering the NIR-I region (808 nm), whereas the maximum absorption peak of pure MB solution was located at 611 and 664 nm. When MB was loaded on HMPB, the peak was observed, which exhibited a slight red shift (667 nm), indicating the interaction between MB and HMPB. After coating Au NPs, PMB and HA, HMA, HMAP and HMAPH all displayed obvious MB peaks (665, 670, and 671 nm). They also exhibited a broad NIR characteristic absorption peak similar to HMPB, suggesting that they could also be used as PTT agents.

Figure 2e shows the crystal structures of HMPB, HMPB@MB, HMA, HMAP and HMAPH analyzed using X-ray diffraction. All diffraction peaks of HMPB matched the diffraction pattern of face-centered cubic Fe^III^_4_[Fe^II^(CN)_6_]_3_ as specified by the Joint Committee on Powder Diffraction Standard Document No. 73–0687 [40]. The diffraction peak of HMPB@MB remained unchanged after MB loading. On further reaction with HAuCl_4_, another diffraction peak appeared at 47.28, corresponding to the (102) plane of Au, proving the successful *in situ* growth of Au particles. After further coating with PMB and HA, the characteristic diffraction peaks of HMPB and Au were still detected. As shown in Figure 2f, the strong absorption band at 2089 cm^−1^ and the sharp peak at 499 cm^−1^ were attributed to the stretching vibration of the C ≡ N bond and the bending vibration of Fe–C ≡ N–Fe in HMPB, respectively [41]. After MB loading, another absorption peak was detected at 1413 cm^−1^ for the C–N bond of MB. As a metal element, Au had no vibrational peaks in the Fourier-transform infrared spectrum, so the infrared spectrum was consistent between HMA and HMPB@MB. We not only observed the stretching vibration of C=N and C–N bonds and the bending vibration of Fe–C ≡ N–Fe but also the stretching vibration of C=O bond in the spectra of HMAP and HMAPH due to the modification of COOH–PEG–SH on the surface of HMA. As shown in the X-ray photoelectron spectra (XPS) in Figure 2g, Fe, O, C, Cl, S and Au elements were present in HMAPH. As shown in Figure 2h, the binding energies at 707.85 and 720.53 eV (2p_3/2_ and 2p_1/2_) represented the fitted peaks of Fe(II), whereas those at 711.55 and 724.20 eV (2p_3/2_ and 2p_1/2_) indicated the fitted peaks of Fe(III) [22]. As shown in Figure 2i, the high-resolution spectra of Au 4f had two peaks at 84.96 and 88.58 eV, corresponding to Au 4f_7/2_ and Au 4f_5/2_, respectively. The C 1 s peaks at 284.9 and 287.9 eV suggested that carbon might exist mainly in the form of C–O and C=O (Figure 2j). As shown in Figure 2k, the binding energies at 164.33, 168.4 and 169.98 eV were ascribed to atomic sulfur, SO_3_^2−^ (2p^3/2^) and SO_3_^2−^ (2p^1/2^), respectively [42,43]. As shown in Figure 2l, the binding energies at 199.57 and 203.70 eV indicated the characteristic peaks of Cl 2p.

### Detection of ROS generation and photothermal performance of HMAPH

**Figure 3:**
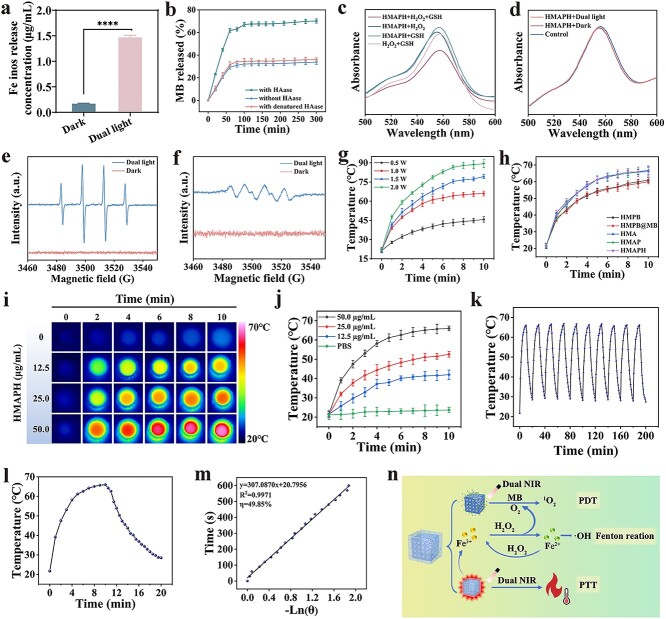
The photothermal effects of the HMAPH. (**a**) Amount of Fe ions released under NIR irradiation of HMAPH. (**b**) Release curve of MB in HMAPH under different conditions. (**c**) Degradation of RhB by HMAPH in different environments under dark conditions. (**d**) Degradation of RhB by HMAPH under dual-light or dark conditions. ESR spectra of HMAPH under dark or light conditions: •OH (**e**) and •O^2−^ (**f**) were detected separately by using BMPO as spin trapping agent. (**g**) Temperature changes of HMAPH (50 μg/ml) under 808 nm NIR irradiation at different powers (0.5, 1.0, 1.5 and 2.0 W/cm^2^). (**h**) Temperature changes of different nanomaterials under 808 nm NIR irradiation (1.0 W/cm^2^). Photothermal images (**i**) and temperature changes (**j**) of different concentrations of HMAPH under 808 nm NIR irradiation (1.0 W/cm^2^). (**k**) Photothermal heating and cooling of HMAPH for 10 cycles. (**l**) ‘On–off’ temperature changes of HMAPH under 808 nm NIR irradiation. (**m**) Photothermal-conversion efficiency of HMAPH under NIR irradiation at 808 nm. (**n**) Schematic illustration of the mechanism of HMAPH for ^1^O_2_ and •OH production and photothermal performance. ^****^p < 0.0001. *NIR* near infrared, *HMAPH* HMPB@MB@AuNPs@PMB@HA, *MB* methylene blue, *RhB* rhodamine B, *ESR* electron spin resonance, *BMPO* 5-tert-butoxycarbonyl 5-methyl-1-pyrroline *N*-oxide, *a.u. *absorbance unit, *PMB* polymyxin b, *HA* hyaluronic acid, *PTT *photothermal therapy, *PDT* photodynamic therapy

As previously reported, Fe ions can react with H_2_O_2_ and other metabolites in bacteria to generate •OH via the Fenton reaction [37]. The release of Fe ions before and after irradiation was measured using inductively coupled plasma mass spectrometry. As shown in Figure 3a, the HMAPH + dual-light irradiation group showed the largest number of Fe ions produced; 8.8-fold higher than that in the control group. Furthermore, the fluctuation of binding energy before and after NIR light irradiation was measured using XPS. In the XPS spectra of Fe 2p before NIR light irradiation, the fitting peaks of Fe (II) were at 707.85 and 720.53 eV (2p_3/2_ and 2p_1/2_), and the fitting peaks of Fe (III) were at 711.55 and 724.20 eV (2p_3/2_ and 2p_1/2_) (Figure 2h). After NIR light irradiation, the Fe (II) fitting peaks were at 707.72 and 720.50 eV (2p_3/2_ and 2p_1/2_), whereas the Fe (III) fitting peaks were at 710.04 and 723.21 eV (2p_3/2_ and 2p_1/2_) (Figure S3a, see online supplementary material). The Fe(II)/Fe(III) ratio increased from 0.42 to 0.73 after NIR light irradiation, indicating that Fe ions in the HMAPH structure were separated and NIR light irradiation caused changes in the ratio of different-valence Fe ions in HMAPH. This was further verified by Figure S3b, where the binding energies of Fe III-(NC) and Fe II-(CN) were 711.55 and 707.85 eV, respectively. Obviously, after NIR light irradiation, the binding energy of Fe III-(NC) and Fe II-(CN) was low, with a more significant shift for Fe III-(NC), indicating that NIR light irradiation resulted in the release of Fe ions from the HMAPH structure by reducing the number of Fe III-(NC) and Fe II-(CN) bonds [22]. This conclusion was further supported by Figure 3a. Subsequently, the amount of MB released through the activation of HAase in HMAPH was detected, and a time–release curve was plotted. As displayed in Figure 3b, 70.19% of MB was released in the presence of HAase within 5 h, compared with only 33.79% in the absence of HAase. HAase was denatured before the release experiment to identify whether it caused the release of MB. As expected, no significant release of MB was detected. This result showed that HMAPH could respond to the on-demand MB release characteristic of HAase.

The effect of HMAPH on oxidative stress in bacteria was investigated by simulating the internal environment of bacteria with H_2_O_2_ and glutathione (GSH). The degradation of rhodamine B (RhB) was tested by adding HMAPH, H_2_O_2_ and GSH into the RhB solution. The degree of degradation of RhB reflected the generation of ROS through the Fenton reaction. As shown in Figure 3c, RhB showed the highest degree of degradation in the presence of H_2_O_2_ and GSH, compared with lower RhB degradation in the presence of HMAPH + GSH, HMAPH + H_2_O_2_, and GSH + H_2_O_2_. These results indicated that the Fe ions released from HMAPH could react with the interior of the cells to generate •OH under dual NIR light irradiation: Fe^3+^ + GSH → Fe^2+^ + GSSG; H_2_O_2_ + Fe^2+^ → Fe^3+^ + OH^−^ + •OH. Furthermore, we tested the ability of HMAPH to produce ROS without the participation of bacteria through RhB to explore whether HMAPH could generate ROS under dual NIR light irradiation. As displayed in Figure 3d, HMAPH could not produce ROS under different conditions without bacteria, confirming that ROS was not produced externally but within bacteria. The aforementioned results were consistent with those reported in the literature [22]. The specific type of ROS governing the oxidase-like activity was explored using 5-*tert*-butoxycarbonyl-5-methyl-1-pyrroline-*N*-oxide (BMPO) and 5,5-dimethyl-1-pyrroline-*N*-oxide as spin-trapping probes for superoxide radical (•O^2−)^ and hydroxyl radical (•OH), respectively. The ROS were measured through electron spin resonance experiments. As shown in Figure 3e and f, HMAPH produced relatively high signals in 1 : 1 : 1 : 1 and 1 : 2 : 2 : 1 ratios under dual NIR light conditions, suggesting that dual-light-induced HMAPH could have higher production of •O^2−^ and •OH.

The photothermal performance of HMAPH was also evaluated. An ideal photothermal agent is expected to have strong absorption in the NIR range of 750–850 nm, which is the transmittance window for biophotothermal therapy. As shown in the UV–vis absorption spectrum of Figure 2d, HMAPH had strong absorption in the NIR region. As expected, the temperature increase in HMAPH was positively correlated with NIR irradiation time and power. As shown in Figure 3g, at 1.0 W/cm^2^ NIR power, the temperature of HMAPH increased to 66°C after 10 min of irradiation. Figure 3h displays the temperature changes of HMPB, HMPB@MB, HMA, HMAP and HMAPH within 10 min of irradiation at 1.0 W/cm^2^. The temperature of HMPB and HMPB@MB increased to about 60°C after exposure to 808-nm laser irradiation for 10 min, compared with an increase to 66°C after 10 min of laser irradiation when Au particles were grown *in situ* on the surface of HMPB, indicating that Au modification improved the photothermal performance of HMPB. Figure 3i and j shows the real-time monitoring of the temperature changes of HMAPH with the increase in sample concentration and irradiation time recorded using a thermal imaging camera. At 50.0 μg/ml HMAPH concentration, the temperature could be increased to 66°C after 10 min of irradiation. As shown in Figure 3k, the heating and cooling curves indicated high photothermal stability of HMAPH. As shown in Figure 3l and m, the photothermal conversion efficiency of HMAPH was 49.85%. HMAPH not only exhibited excellent photothermal conversion efficiency at low power densities but also demonstrated sustained photothermal cycling stability compared with other photothermal nanomaterials in the same field, such as bioactive glass material Ce-BG, MOF material HMIL-ACF-Por and precious metal nanomaterial SiO_2_@AuAg/PDA [44–46].

Overall, HMAPH accelerated the release of Fe ions due to its high photothermal conversion performance, and the released Fe ions catalyzed the conversion of H_2_O_2_ into •OH under dual-light irradiation conditions. Additionally, the photodynamic activity was also triggered to generate superoxide anion radicals due to the encapsulation of photosensitizer MB (Figure 3n).

### Evaluation of *in vitro* antibacterial activity

**Figure 4:**
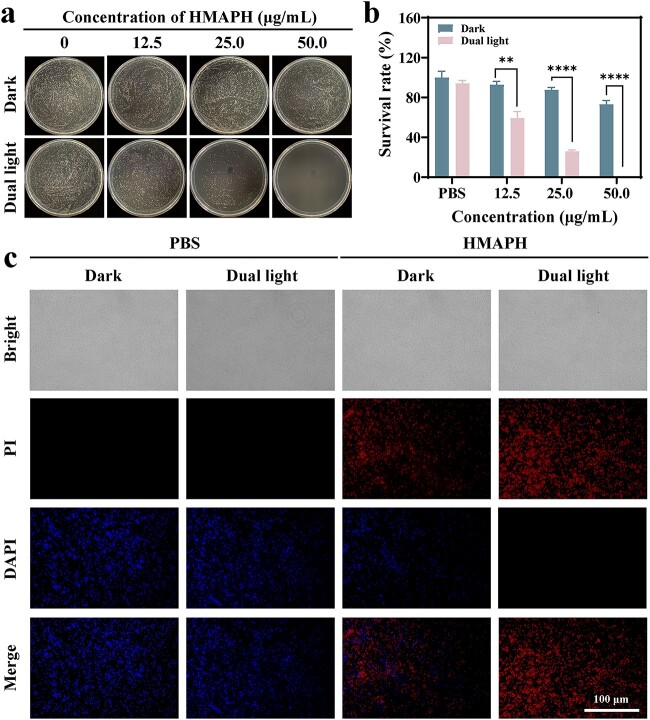
Photographs of colonies formed by *Pseudomonas aeruginosa* (*P. aeruginosa*) treated with different concentrations of HMAPH under dark conditions and dual-light irradiation for 10 min (**a**) and the corresponding antibacterial rates of *P. aeruginosa* (**b**). (**c**) Fluorescence images of *P. aeruginosa* stained with DAPI (blue) and PI (red) after treatment under different conditions; scale bar: 100 μm. ^**^p< 0.01, ^****^p < 0.0001. *HMAPH* HMPB@MB@AuNPs@PMB@HA, *PI* propidium iodine, *DAPI* 4′,6-diamidino-2-phenylindole, *PMB* polymyxin b, *HA* hyaluronic acid

Inspired by its satisfactory photothermal properties and tunable generation of ^1^O_2_ and •OH radicals, the antibacterial properties of HMAPH against *P. aeruginosa* were evaluated through *in vitro* experiments. The antibacterial rates of various concentrations of HMAPH (12.5, 25 and 50 μg/ml) under different NIR irradiation are shown in Figure 4a and b. No antibacterial effect was detected in the control group, regardless of light exposure, suggesting that light alone had no effect on bacterial growth. Under dual-light irradiation, the antibacterial effect of HMAPH increased with the increase in HMAPH concentration. Meanwhile, we measured the antibacterial activity of different materials (HMPB, HMPB@MB, HMA and HMAPH) at the same concentration (50 μg/ml). The experimental results showed that the antibacterial rates against *P. aeruginosa* were 94.60, 96.71, 99.89 and 99.97% for HMPB, HMPB@MB, HMA and HMAPH under dual light, respectively (Figure S4, see online supplementary material). The inhibition rate of HMAPH was higher than that in other groups, indicating that HMAPH displayed good antibacterial activity under the synergistic effect of PTT, PDT and self-induced Fenton reaction. Moreover, the antimicrobial performance of HMAPH against *S. aureus* was evaluated (Figure S5, see online supplementary material). The survival rate of *S. aureus* after 50 μg/ml HMAPH + dual-light treatment was 13.7%. In contrast, the survival rate of *P. aeruginosa* was as low as 0.03%, which further proved the targeting of HMAPH against gram-negative bacteria.

The antibacterial ability of HMAPH was further investigated using live/dead fluorescence tests, with live bacteria stained blue and dead bacteria stained red. As shown in Figure 4c, the control *P. aeruginosa* exhibited blue fluorescence emission and no red spots on the surface under light or dark conditions, indicating good bacterial growth. Conversely, the surface of bacteria treated with 50.0 μg/ml HMAPH showed partial red fluorescence, indicating that HMAPH alone had a certain antibacterial activity. When treated with HMAPH + dual light, the surface of the bacteria was almost all red, demonstrating the death of almost all the bacteria. These results are consistent with the findings of agar plate experiments, suggesting the antibacterial efficacy of the synergistic system.

**Figure 5:**
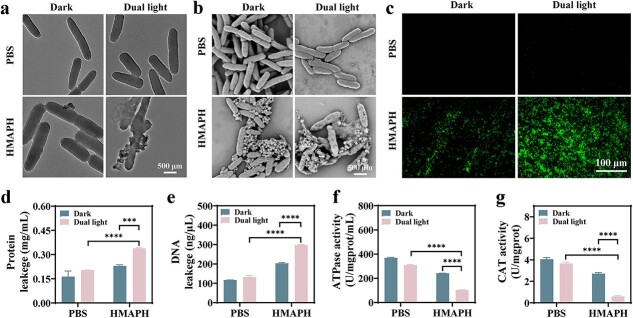
Antibacterial mechanism of HMAPH. TEM (**a**) and SEM (**b**) images of *Pseudomonas aeruginosa (*P. aeruginosa*)* after HMAPH treatment under different conditions. (**c**) Fluorescence images of ROS in *P. aeruginosa* under different HMAPH treatment conditions and stained green with DCFH-DA; scale bar: 100 μm. The leakage of protein (**d**) and DNA (**e**) after HMAPH treatment under dual light or dark conditions. ATPase (**f**) and CAT (**g**) activity in *P. aeruginosa* after HMAPH treatment under dual-light or dark conditions. ^***^p < 0.001, ^****^p < 0.0001. *TEM* transmission electron microscope, *SEM* scanning electron microscope, *HMAPH* HMPB@MB@AuNPs@PMB@HA, *ROS* reactive oxygen species, *DCFH-DA* 2′,7’-dichlorodihydrofluorescein diacetate, *DNA* deoxyribonucleic acid, *ATPase* adenosine triphosphatase, *CAT* catalase

The morphology and membrane integrity of *P. aeruginosa* were investigated using TEM. As shown in Figure 5a, the control bacterial membranes exhibited smooth and intact morphology under dark or dual light for 10 min. The cell membrane was intact and smooth after incubation of HMAPH and *P. aeruginosa* in the dark for 10 min, indicating that HMAPH was almost nontoxic to bacteria. However, the integrity of the bacteria was destroyed after 10 min of dual-light irradiation, resulting in severe rupture of bacterial membranes. As shown in Figure 5b, the conclusion of scanning electron microscopy (SEM) characterization was the same as that of TEM analysis. Both SEM and TEM results demonstrated the recognition properties of HMAPH for *P. aeruginosa*. Additionally, in *Staphylococcus aureus* and *P. aeruginosa* mixed suspensions cultured with HMAPH, only *P. aeruginosa* cells rather than *S. aureus* cells were surrounded by HMAPH (Figure S6, see online supplementary material). When *P. aeruginosa* was treated with HMAPH under any conditions, a large number of HMAPH NPs were present on the bacterial surface. This was mainly because the modified PMB targeted and bound to the outer membrane of gram-negative bacteria, allowing better enrichment of HMAPH on the bacterial surface. After 10 min of dual-light irradiation, intracellular cytoplasmic efflux was generated due to the degeneration and rupture of the cell walls of *P. aeruginosa*. We further quantified the protein and DNA efflux of *P. aeruginosa* under different conditions (dark and dual light). As shown in Figure 5d and e, the leakage of protein and DNA increased to 0.339 ± 0.003 mg/ml (*vs* 0.164 ± 0.034 mg/ml) and 297.51 ± 2.23 ng/μl (*vs* 116.66 ± 0.63 ng/μl), respectively, under HMAPH + dual-light treatment, further proving the antibacterial advantage of the synergistic system.

The aforementioned results indicated the successful establishment of the synergistic antibacterial platform of PDT, self-induced Fenton reaction and PTT. Whether the antibacterial efficiency of HMAPH was partly attributed to ROS was verified by 2′,7′-dichlorodihydrofluorescein diacetate staining to further confirm ROS production under different treatment conditions. As shown in Figure 5c, the control group showed no green fluorescence signal under either dark or light conditions. However, strong green fluorescence was observed under the dual-light treatment, proving the combined effect of PDT and PTT sterilization. ROS-mediated oxidative stress was found to impair bacterial metabolism and disrupt bacterial homeostasis [47]. As shown in Figures 5f, 5g and S7 (see online supplementary material), ATPase, catalase and GSH levels significantly decreased in *P. aeruginosa* after HMAPH + dual-light treatment. Overall, HMAPH exhibited an antibacterial effect under dual-light irradiation, which was because PTT could alter the permeability of bacterial membranes and reduce bacterial activity. However, ROS-mediated oxidative stress could impair bacterial metabolism, disrupt membrane structure and ultimately synergistically cause cell death.

### *In vitro* anti-biofilm activity and quorum sensing inhibition ability

**Figure 6:**
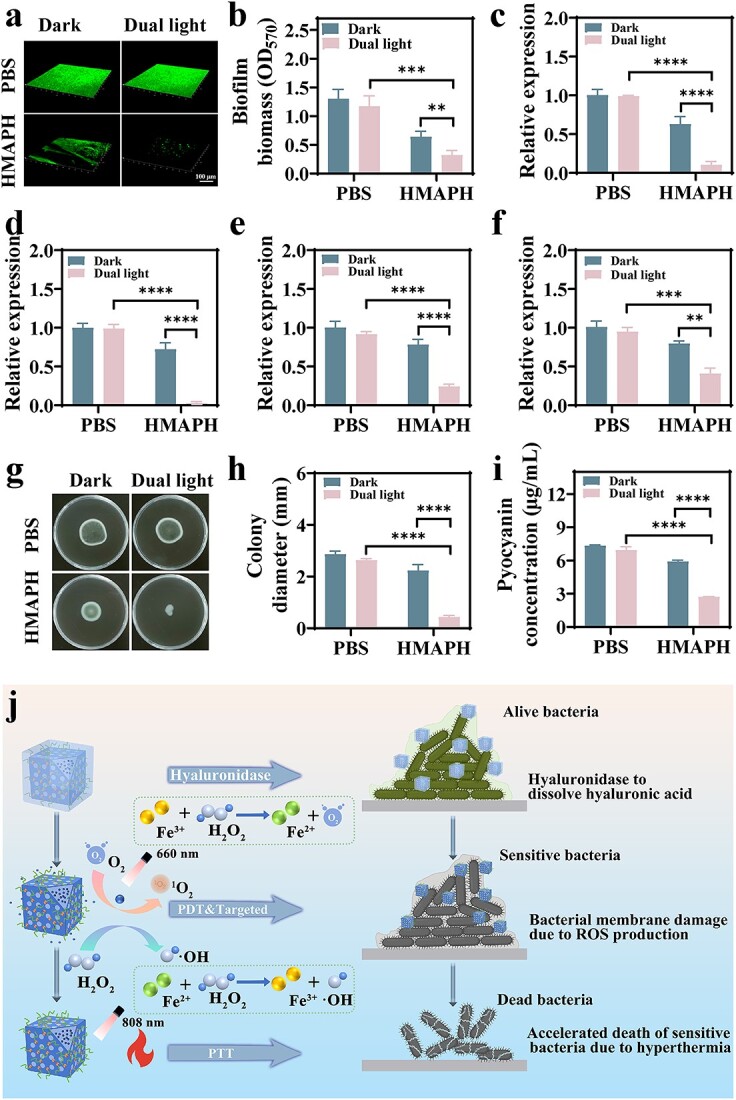
Antibiofilm properties of HMAPH. (**a**) 3D images of Pseudomonas aeruginosa (*P. aeruginosa)* biofilms after HMAPH treatment under dual-light or dark conditions. Scale bar: 100 μm. (**b**) Biofilm biomass after HMAPH treatment under dual-light or dark conditions. RT-qPCR analysis of the relative expression levels of *lasB* (**c**), *lasR* (**d**), *rhlR* (**e**) and *pilG* (**f**) of *P. Aeruginosa*. Floating motion plate images (**g**) and corresponding colony diameter (**h**) after different treatments. (**i**) Content of pyocyanin after different treatments. (**j**) Biofilm removal mechanism of HMAPH under dual-light irradiation. ^**^p < 0.01, ^***^p < 0.001, ^****^p < 0.0001. *HMAPH* HMPB@MB@AuNPs@PMB@HA, *RT-qPCR* reverse transcription-polymerase chain reaction, *MB* methylene blue, *PMB* polymyxin b, *HA* hyaluronic acid, •OH hydroxyl radical,*P. aeruginosa* Pseudomonas aerginosa, *ROS *reactive oxygen species, *PTT* photothermal therapy, *PBS* phosphate-buffered saline

The anti-biofilm activity of HMAPH was systematically evaluated by crystal violet and SYBR Green I staining. *Pseudomonas aeruginosa* biofilms were treated with HMAPH (50 μg/ml) under dual-light irradiation or dark conditions. As shown in Figure 6b, the HMAPH + dual light irradiation group showed obviously lower OD_570_ values compared with the control PBS group, suggesting the inhibition of biofilm formation. Figure 6a presents the 3D images of SYBR Green I-stained biofilms, visually reflecting the inhibitory and disruptive effects of different treatments on biofilms. Of the four groups, the HMAPH + dual-light irradiation group had the thinnest biofilm and the weakest fluorescence intensity.

Quorum sensing is a mechanism by which pathogenic bacteria regulate the production of their virulence factors, thereby exacerbating the disease [48]. *Pseudomonas aeruginosa* can produce various virulence factors that aid in the pathogenic process by helping bacteria in colonizing adherent surfaces and degrading immune proteins to interfere with the host immune system [49]. Whether HMAPH might affect the expression of *P. aeruginosa* virulence factors was evaluated by RT-qPCR of the expression levels of virulence genes (*lasB*, *lasR*, *rhlR* and *pilG*). As shown in Figure 6c–f, the four genes (*lasB*, *lasR*, *rhlR* and *pilG*) were significantly downregulated after HMAPH + dual-light treatment. Additionally, motility is considered as one of the important virulence factors for biofilm formation. Therefore, we further evaluated whether HMAPH could inhibit the motility of *P. aeruginosa*. As shown in Figure 6g, HMAPH + dual-light treatment markedly suppressed the movement of *P. aeruginosa* on the surface of the plate compared with the blank control treatment. As shown in Figure 6h, the diameter of floating movement distinctly reduced from 2.87 ± 0.12 mm to 0.43 ± 0.06 mm (*P* < 0.0001). Additionally, pyocyanin, another important virulence factor activated by quorum sensing, is a blue-green secondary metabolite produced by *P. aeruginosa*, which can catalyze the decomposition of superoxide or peroxide to produce toxic oxidative groups, thereby causing tissue damage [50]. As shown in Figure S8, see online supplementary material, the test tubes had the deepest color in the control group and the lightest color in the HMAPH + dual-light irradiation group. As shown in Figure 6i, quantitative analysis showed that the pyocyanin content decreased from 7.35 ± 0.05 to 2.72 ± 0.02 μg/ml (*P* < 0.0001), indicating that HMAPH could inhibit the synthesis of pyocyanin under dual-light irradiation.

Based on the aforementioned results, we proposed the mechanism of photo-induced biofilm removal (Figure 6j). The HAase secreted by *P. aeruginosa* degraded the HA wrapped on the surface of HMAPH, enabling the specific binding of exposed nanomaterials to the outer membrane of *P. aeruginosa* through PMB. This led to the instability of the bacterial outer membrane, thus enhancing the sensitivity of gram-negative bacteria to PDT. Under dual-light irradiation (808 + 660 nm), HMAPH exhibited a high photothermal effect. Also, the local high temperature could accelerate the release of Fe ions, triggering ROS generation via the Fenton reaction. Meanwhile, 660-nm light irradiation also induced ROS generation by photosensitizer MB. Generally, ROS-mediated oxidative stress can disrupt bacterial metabolism, and the photothermal effect can easily alter the permeability of bacterial cell membranes and denature proteins and enzymes in them. Therefore, the synergy of PTT, PDT and self-induced Fenton reaction might be an ideal approach to eliminate biofilms.

### Evaluation of *in vivo* antibacterial activity

The application prospects for HMAPH in wound disinfection and healing was further investigated. Before treatment, the safety of HMAPH was assessed as a prerequisite. After injecting the mice with HMAPH (200 μl, 50.0 μg/ml) for 48 h, acute toxicity was evaluated using serum biochemical tests and hematoxylin and eosin (H&E) staining of major visceral organs. As shown in Figure S9a–f (see online supplementary material), the HMAPH-injected mice showed no significant difference compared with the healthy mice in the levels of alanine transaminase (ALT), aspartate aminotransferase (AST), alkaline phosphatase (ALP), albumin (ALB), lactate dehydrogenase (LDH) and creatinine (Cr). This suggested no significant toxicity for HMAPH at a given dose. H&E staining revealed no significant difference in the internal organs (heart, liver, spleen, lung and kidney) between the HMAPH-treated and blank groups (Figure S9g). These results demonstrated that HMAPH (200 μl, 50 μg/ml) could be safely used in the subsequent animal experiments.

**Figure 7:**
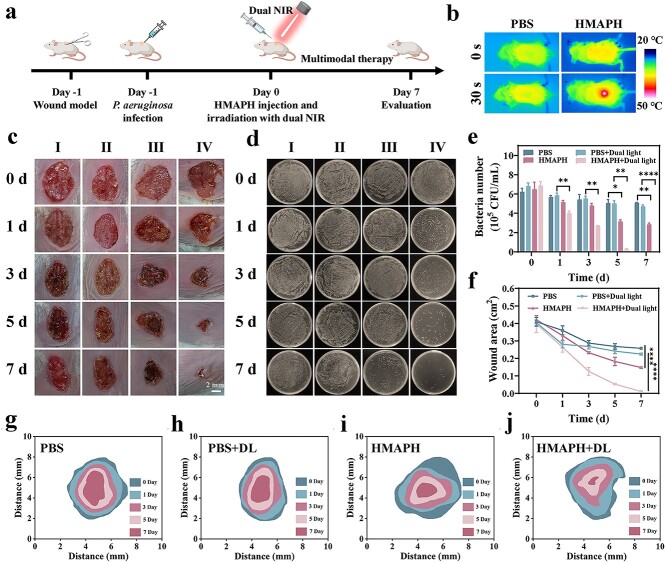
*In vivo* wound healing assay. (**a**) Schematic diagram of anti-*Pseudomonas aeruginosa* infection treatment. (**b**) Thermal imaging of mouse abscesses before and after irradiation for 30 s in different treatment groups. (**c**) Photographs of *P. aeruginosa*-infected wounds of mice in various treatment groups within 7 days of treatment. Groups I–IV: (I) PBS, (II) PBS + dual light, (III) HMAPH, (IV) HMAPH + dual light; scale bar: 2.0 mm. (**d**) Photographs of bacterial colonies in wounds in various treatment groups. (**e**) Number of viable bacteria in wound tissue of different groups was counted on day 1, 3, 5 and 7 of treatment. (**f**) Quantitative analysis of wound area of mice after various treatments. Dynamic process of wound healing within 7 days in groups of PBS (**g**), PBS + dual light (**h**), HMAPH (**i**) and HMAPH+dual light (**j**). ^*^p < 0.05, ^**^p < 0.01, ^****^p < 0.0001. *HMAPH* HMPB@MB@AuNPs@PMB@HA, *PBS* phosphate-buffered saline, *CFU *colony forming units, *MB* methylene blue, *PMB* polymyxin b, *HA* hyaluronic acid, *NIR* near infrared

A mouse model of skin wound infected by *P. aeruginosa* was established to evaluate the antibacterial activity of HMAPH (Figure 7a). The infected mice were randomly divided into four groups: (I) PBS, (II) PBS + dual light, (III) HMAPH and (IV) HMAPH + dual light. One day after successful infection, the mice were treated as indicated for the different groups. Wound diameters and photographs were recorded every 2 days, and the bacteria on the surface of each wound were quantified. As shown in Figure 7b, the temperature of the wound increased in the HMAPH-treated groups after 30 s of dual-light irradiation. The wound temperature increased to 48.72°C, which was obviously higher than the wound temperature in the PBS group (38.28°C). As shown in Figure 7c, the wounds in the control group developed obvious erythema and inflammation during treatment. However, the wounds of the mice in the HMAPH + dual-light irradiation groups began to scab after 3 days of treatment, and the wound area decreased significantly after 5 days of treatment. After 7 days of HMAPH + dual-light treatment, the wounds were essentially healed. Wound skin samples were collected from each group after treatment and the wound area was measured. Bacterial counts were determined in each group of wounds using the plate method to quantitatively assess the antibacterial effect of each treatment. As shown in Figure 7f, the wound healing rates were 37.16, 44.56 and 65.08% in groups I–III, respectively, after 7 days of treatment. HMAPH achieved superior wound healing results in a shorter timeframe compared with other similar photothermal nanomaterials, e.g. the polymer-based nanomaterial CPAP/PDA@Cu [51], MOF material MN-MOF-GO-Ag [52] and noble metal nanomaterial PHMB@Au NPs [53]; the wound healing rate was as high as 97.35%. Figure 7g–j shows the simulation diagrams of the dynamic tracking of wound area in different treatment groups within 7 days. As shown in Figure 7d and e, the number of bacteria was less in the HMAPH + dual-light irradiation group than in the other groups after 7 days of treatment, indicating that HMAPH + dual light had the strongest bactericidal effect.

We added commercial dressings containing silver ions (Shenzhen Yuanxing Pharmaceutical Co., Ltd, China) as a positive control to demonstrate the clinical applicability of HMAPH. As shown in Figure S10 (see online supplementary material), silver-ion-containing commercial dressings effectively inhibited wound infection and promoted wound healing. The bacterial inhibition rate and wound healing rate in the silver-ion-containing commercial dressing group was 91.56 and 90.68%, respectively. In contrast, the bacterial inhibition rate and wound healing rate in the HMAPH + dual-light irradiation group were up to 99.90 and 97.35%, respectively. These results indicated that HMAPH had broad application prospects as a potential antibacterial agent.

**Figure 8:**
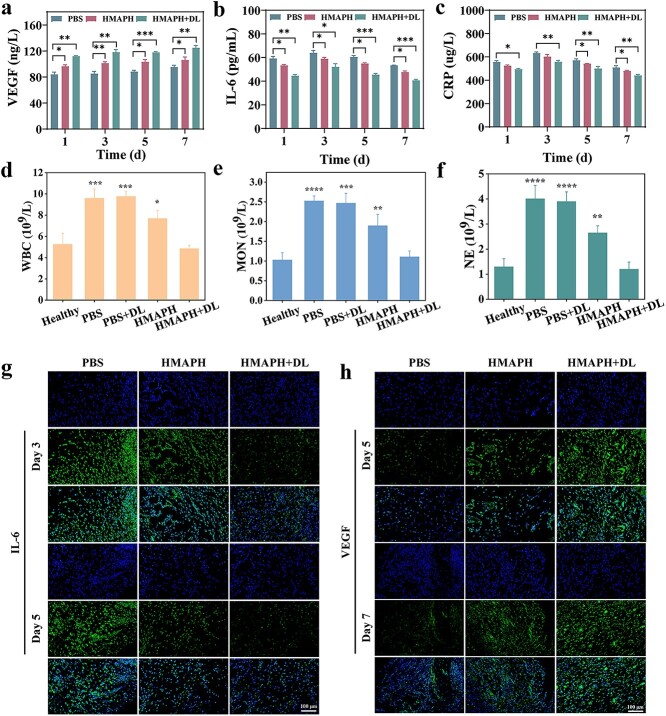
Inflammation-related investigations in serum and tissues in wound healing. Changes in VEGF (**a**), IL-6 (**b**) and CRP (**c**) levels in serum at different time points during treatment in different treatment groups. Changes in white blood cell (WBC) (**d**), monocyte (MON) (**e**) and neutrophil (NE) (**f**) counts in different treatment groups on day 7. Image of immunofluorescent staining of regenerated wound tissues labeled with (**g**) IL-6 on days 3 and 5 and (**h**) VEGF on days 5 and 7; scale bar: 100 μm. ^*^p < 0.05, ^**^p < 0.01, ^***^p < 0.001, ^****^p < 0.0001. *HMAPH* HMPB@MB@AuNPs@PMB@HA, *VEGF* vascular endothelial growth factor, *IL-6* interleukin 6, *CRP *c-reactive protein, *PBS* phosphate-buffered saline, *MB* methylene blue, *PMB* polymyxin b, *HA* hyaluronic acid

Additionally, interleukin 6 (IL-6) and vascular endothelial growth factor (VEGF) were used to further investigate the efficacy of HMAPH treatment in the wound healing process. The levels of IL-6 and VEGF in the serum were quantitatively measured at different time points during the treatment of *P. aeruginosa* infection in different groups. As shown in Figure 8a, the HMAPH treatment group displayed an increasing trend in VEGF level with an increase in treatment time, especially on day 7 of treatment, with a significantly higher VEGF level in the HMAPH + dual-light irradiation group (124.96 ± 3.77 ng/l) than in the PBS group (95.38 ± 2.72 ng/l). As shown in Figure 8b, IL-6 reached the highest level in the HMAPH and HMAPH + dual-light treatment groups on day 3 of *P. aeruginosa* infection and then gradually reduced and was significantly lower than the level in the PBS group on day 7. As expected, the amount of C-reactive protein in mouse serum was also significantly lower on day 7 in mice treated with HMAPH + dual light compared with PBS treatment, indicating the recovery of bacterial infection (Figure 8c). Moreover, we further analyzed the degree of bacterial infection by measuring blood routine, such as white blood cell, monocyte and neutrophil counts. As shown in Figure 8d–f, HMAPH + dual light reduced white blood cell, monocyte and neutrophil counts to nearly the levels in the healthy mice compared with PBS + dual light, suggesting that infection and inflammation in the mice could be effectively eliminated after HMAPH + dual-light treatment. Figure 8g and h shows the expression of IL-6 and VEGF detected using immunofluorescence staining during the treatment. As shown in Figures 8g and S11a (see online supplementary material), IL-6 expression decreased on days 3 and 5 in the HMAPH and HMAPH + dual-light irradiation groups compared with the PBS group. The quantitative results showed that the inhibitory effect of HMAPH + dual light was more significant (*P* < 0.01). As shown in Figures 8h and S11b, the expression of VEGF gradually increased on days 5 and 7 of treatment. The results showed that, among the three groups, the HMAPH + dual-light irradiation group had the highest expression level of VEGF, with an extremely significant difference between the HMAPH + dual-light irradiation and PBS groups (*P* < 0.001). These results indicated that HMAPH could promote *P. aeruginosa-*infected wound healing by downregulating the levels of inflammatory cytokines and upregulating the levels of VEGF under dual-light irradiation.

**Figure 9:**
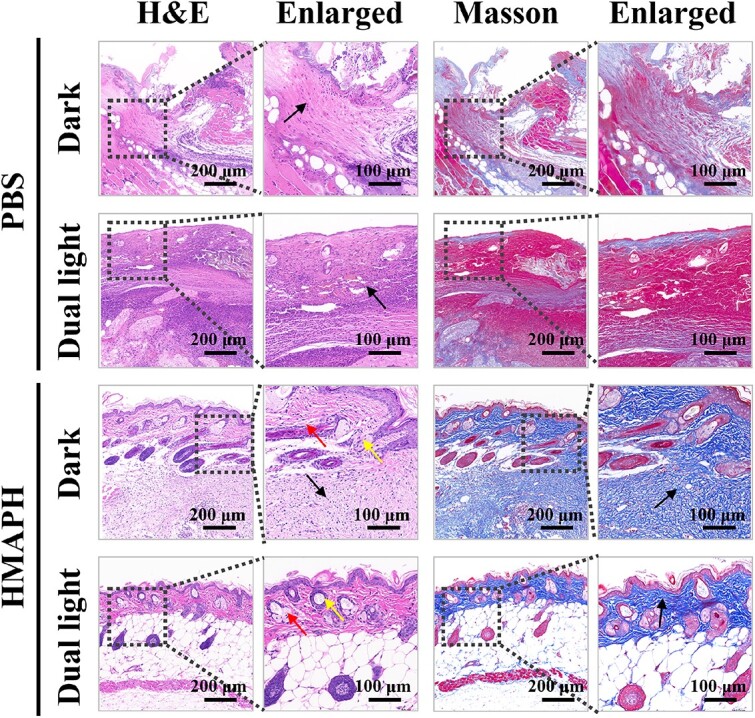
H&E and Masson staining images of wound tissues treated with PBS, PBS + dual light, HMAPH and HMAPH + dual light on day 7 of treatment, respectively. *HMAPH* HMPB@MB@AuNPs@PMB@HA, *PBS* phosphate-buffered saline, *MB* methylene blue, *PMB* polymyxin b, *HA* hyaluronic acid,* H&E *hematoxylin & eosin

Additionally, the wound tissue sections of mice in different treatment groups were stained with H&E and Masson’s trichrome for histological analysis. As shown in Figure 9, the mice in the PBS and PBS + dual-light irradiation groups had a severely abnormal skin structure, with massive inflammatory cell infiltration (indicated by black arrows). However, the mice in the HMAPH group showed significant improvement in the degree of damage compared with the PBS group, with relatively intact epidermis and dermis, skin accessory organs in the dermis, hair follicles (shown by yellow arrows), sebaceous glands (shown by red arrows) and infiltration of a small number of inflammatory cells (shown by black arrows). Meanwhile, the mice in the HMAPH + dual-light irradiation group showed basically normal skin structure, with a clear structure of epidermis and dermis and no obvious pathological changes. Accessory organs were visible in the dermis, with red arrows for sebaceous glands and yellow arrows for hair follicles. Masson staining results revealed the absence of a large number of collagen fibers in the PBS group, compared with a significant increase in the number of collagen fibers in the HMAPH groups. Especially in the HMAPH + dual-light irradiation group, the collagen of wound tissue was more abundant and neatly arranged, suggesting that its wound repair effect was better than the other treatments.

**Figure 10:**
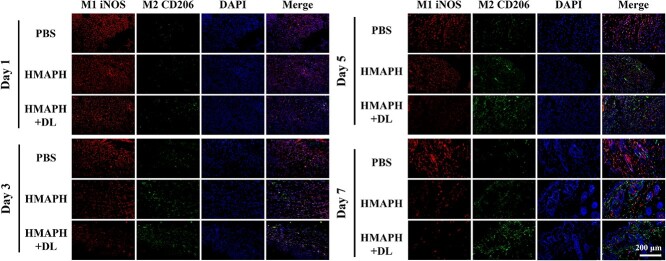
Immunofluorescence staining pictures of M1 (red) and M2 (green) macrophages in wound tissues of different treatment groups on day 1, 3, 5, and 7 of treatment; scale bar: 100 μm. *HMAPH* HMPB@MB@AuNPs@PMB@HA, *PBS* phosphate-buffered saline, *DAPI* 4′,6-diamidino-2-phenylindole, *iNOS* inducible nitric oxide synthase, *MB* methylene blue, *PMB* polymyxin b, *HA* hyaluronic acid

Meanwhile, the changes of M1 (red) and M2 (green) macrophages in the skin of mice on days 1, 3, 5 and 7 were assessed by immunofluorescence staining. As shown in Figure 10, the proportion of M1 cells in mice tissues increased in the early stage of infection, suggesting that macrophages mainly differentiated into M1 macrophages and released pro-inflammatory factors (IL-1, IL-6, ROS, etc.) to kill bacteria [54,55]. Clearly, the proportion of M1 macrophages in tissues significantly reduced after HMAPH + dual-light treatment, leading to earlier polarization of M2 macrophages. Especially, the mice in the PBS group showed significantly more red fluorescence than green fluorescence on day 7, implying the differentiation of M1 macrophages. However, HMAPH treatment induced a decrease in iNOS expression and an increase in CD206 expression, showing the polarization of M1 macrophages toward M2, which helped reduce inflammation and promote tissue damage repair. The degree of M2 polarization of macrophages was higher in the HMAPH + dual-light irradiation group compared with the HMAPH group (Figure S11c, see online supplementary material). These results demonstrated that HMAPH affected bacterial inhibition and injury recovery *in vivo* and might serve as a potential targeted antibacterial agent against infections caused by gram-negative bacteria.

### Biosafety assay

**Figure 11:**
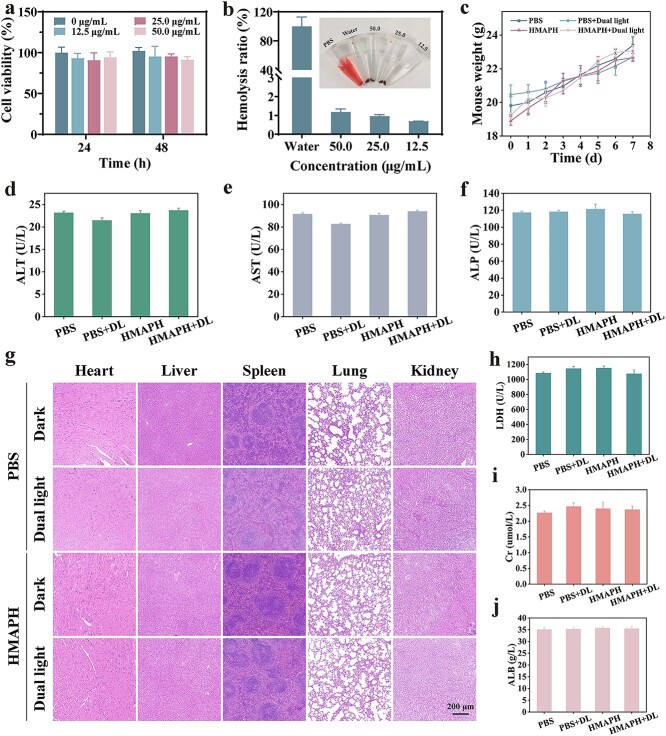
Biosafety experiments of HMAPH. (**a**) Cell viability after incubation with various concentrations of HMAPH for 24 and 48 h. (**b**) Hemolysis rates at different concentrations of HMAPH (inset presents photographs of hemolysis reactions in different groups). PBS and deionized H_2_O were utilized as negative control and positive control, respectively. (**c**) Body weight of mice during treatment in different groups. Blood biochemical indicators for different groups related to liver and kidney functions, including alanine transaminase (ALT) (**d**), aspartate aminotransferase (AST) (**e**), alkaline phosphatase (ALP) (**f**), lactate dehydrogenase (LDH) (**h**), creatinine (Cr) (**i**) and albumin (ALB) (**j**). (**g**) H&E staining of major organs (heart, liver, spleen, lung, kidney) in various treatment groups on day 7 of treatment, scale bar: 200 μm. *HMAPH* HMPB@MB@AuNPs@PMB@HA, *H&E* hematoxylin & eosin, *PBS* phosphate-buffered saline, *MB* methylene blue, *PMB* polymyxin b, *HA *hyaluronic acid

For safety reasons, the potential toxicity of HMAPH was evaluated *in vitro* and *in vivo*. Different concentrations of HMAPH and NIH-3 T3 cells were co-incubated separately for 24 and 48 h, followed by analysis of the cytotoxicity using 3-(4,5-Dimethylthiazol-2-yl)-2,5-dipheOKnyltetrazolium bromide (MTT). In Figure 11a, the relative survival rate of cells treated with different concentrations of HMAPH was >90%, indicating good biocompatibility of HMAPH. As shown in Figure 11b, the hemocompatibility of HMAPH was measured using the hemolysis test. After incubation with the highest concentration of HMAPH (50 μg/ml), the hemolysis rate was 1.17 ± 0.17%, significantly below the threshold value of 5.0%. We also investigated the safety of HMAPH and its effect on the migratory ability of fibroblasts through cell migration experiments. Fibroblasts are cells involved in tissue repair processes. As shown in Figure S12 (see online supplementary material), HMAPH treatment could successfully promote the migration of cells compared with PBS treatment, and the migration rate was positively correlated with the material concentration and incubation time.

Mouse body weight was recorded daily during the wound healing experiment. As shown in Figure 11c, body weight displayed no significant difference between different treatment groups. Besides, blood biochemical indicators were assessed after sacrificing mice on day 7 of treatment (Figure 11d–j). The treatment and PBS groups showed no significant difference in liver function indicators (alanine transaminase, aspartate aminotransferase, alkaline phosphatase, albumin and lactate dehydrogenase) and kidney function indicators (Cr), indicating that HMAPH treatment had little effect on the normal functioning of the liver and kidney. As shown in Figure 11g, H&E staining displayed no significant changes in the histological structure of major organs (heart, liver, spleen, lung and kidney) between the treatment and PBS groups. All *in vitro* and *in vivo* experimental results proved the biocompatibility and biosafety of HMAPH.

## Discussion

Bacterial infections, especially those triggered during the wound healing process, have become a serious public health concern globally [56]. *Pseudomonas aeruginosa* has been prioritized by the World Health Organization as a key pathogen for research due to its natural resistance and biofilm-forming ability [57]. The widespread use of conventional antibiotics has triggered an increase in drug-resistant strains and new antimicrobial therapeutics urgently need to be developed to address this challenge. The infections caused by *P. aeruginosa* have become increasingly problematic in clinical settings, primarily due to its biofilm-forming ability and multi-drug resistance. Conventional antibiotic therapies are ineffective against these drug-resistant strains, making the development of new therapeutic strategies particularly important. In this context, the application of nanomaterial-based PDT, PTT and their combination strategies in antimicrobial therapy has attracted much attention.

Photothermal sterilization relies on photothermal conversion materials, such as Au NPs [58], carbon nanomaterials [59] and semiconductor NPs [60], which absorb specific wavelengths of light (usually NIR) and convert it into heat energy. This heat energy kills the bacteria directly. High temperatures can have various detrimental effects on bacterial cells; these include damaging the integrity of the bacterial cell membranes, resulting in membrane rupture and leakage of cellular contents, ultimately leading to bacterial death. Additionally, high temperatures can induce protein denaturation, causing the unfolding of protein molecules and loss of their natural functions. This disrupts bacterial metabolism and structural integrity. Further, DNA and RNA damage can occur, where high temperatures directly cause breaks in bacterial nucleic acid molecules, base mismatches and other changes, affecting the transmission of genetic information and normal physiological functions of bacteria.

Photodynamic sterilization, in contrast, relies on photosensitizers such as MB or other organic dyes, which are activated by light irradiation at specific wavelengths to produce ROS, including singlet oxygen (^1^O_2_), superoxide anion (O^2−^), and hydroxyl radicals (•OH). These ROS are strong oxidants capable of damaging bacterial cell membranes, proteins and DNA, ultimately causing bacterial death. PDT destroys bacterial cell membranes and DNA by inducing ROS production, leading to increased permeability of bacterial cell membranes, leakage of cell contents and bacterial death. In addition, ROS can oxidize the key biomolecules of bacteria, disrupting their normal physiological functions [61,62].

Compared with the Au nanorod-based PTT system studied by Li *et al*. [63], which might damage normal tissues at high temperatures, the introduction of the Fenton reaction and PDT in our system could achieve highly efficient bactericidal effects at lower temperatures, thus reducing damage to surrounding normal tissues. The combination therapy of PDT and PTT introduced by Liu *et al*. [30] failed to use the Fenton reaction to further enhance the sterilization effect, despite its excellent performance under low-dose and low-power laser conditions. Our study further optimized the therapeutic effect compared with other studies on existing combination therapies. Compared with Xie *et al*. [37], we introduced the Fenton reaction, which enabled effective sterilization even under low-H_2_O_2_ conditions, and combined dual illumination with PDT and PTT to enhance overall antimicrobial efficiency. Compared with the combination of nanocatalysis and PTT used by Hu *et al*. [36], our triple combination system significantly enhanced the penetration and sterilization of biofilms through the synergistic effects of multiple mechanisms.

The experimental results showed that our HMAPH could kill 99.97% of *P. aeruginosa* and effectively remove biofilms in 10 min under dual-light irradiation. This result verified the superiority of the triple combination system. Future studies can further optimize the biocompatibility and safety of the nanomaterials and develop a more intelligent light control system for more precise and efficient treatment of bacterial infections.

## Conclusions

In this work, we developed a multifunctional specific therapeutic photo-activated release nanosystem (HMAPH) by loading photosensitizer MB into HMPB nanostructures, followed by modifying the surface sequentially with gold particles, PMB and hydrophilic HA. Upon the injection of HMAPH into the bacterial infection site, HAase will first decompose HA on the surface, allowing the exposed nanomaterials to specifically bind the outer membrane of *P. aeruginosa* through PMB, leading to instability of the bacterial outer membrane to trigger MB release under light and initiate the photodynamic effect to generate ROS. Additionally, the slightly acidic environment at the bacterial infection site and the photothermal effect under light irradiation will accelerate HMPB degradation to release iron ions to induce the Fenton reaction, and the resulting ROS and lipid oxidation will lead to cell damage and death. More importantly, the intrinsic photothermal conversion characteristics of HMPB and the increased photothermal effect of the surface modified with gold particles greatly improved antibacterial efficacy. Meanwhile, the ROS generated by photosensitizer MB and the self-induced Fenton reaction synergized with the photothermal effect and exhibited a strong effect on bacterial infection inhibition and biofilm clearance. Further *in vivo* experiments revealed that HMAPH can shorten the M1 polarization cycle, induce the premature polarization of macrophages into M2 macrophages, promote the secretion of growth factors and enhance wound healing. Overall, *in vitro* and *in vivo* experiments demonstrated that the synergistic HMAPH nanosystem can ameliorate the difficulties with traditional therapeutic approaches, providing a promising strategy for effectively removing biofilms and promoting wound healing.

## Abbreviations

GSH: Glutathione; HA: Hyaluronic acid; HAase: Hyaluronidase; H&E: Hematoxylin & eosin; HMA: HMPB@MB@AuNPs; HMAP: HMPB@MB@AuNPs@PMB; HMAPH: HMPB@MB@AuNPs@PMB@HA; HMPB: Hollow mesoporous Prussian blue; IL-6: Interleukin 6; MD: Methylene blue; MOF: Metal–organic framework; NIR: Near-infrared; NP: Nanoparticle; PMB: Polymyxin B; PB: Prussian blue; PBS: Phosphate-buffered saline; PTT: Photothermal therapy; PDT: Photodynamic therapy; PVP: Polyvinyl pyrrolidone; RhB: Rhodamine B; ROS: Reactive oxygen species; SEM: Scanning electron microscopy; TEM: Transmission electron microscopy; VEGF: Vascular endothelial growth factor; XPS: X-Ray photoelectron spectroscopy.

## Ethics approval and consent to participate

All the animal experiments were approved by the animal care and experiment committee of Tianjin University of Science & Technology (20221006).

## Supplementary Material

Supplementary_material_tkae038

## References

[1] Xu QL , Zhu P, Zhang JR, Liu YH, Cai L, Jiang HJ. et al. Electrochemical formation of distinct nanostructured MoS_2_ with altered antibacterial activity. *Mater Lett* 2020;271:127809. 10.1016/j.matlet.2020.127809.

[2] Cai L , Huang YQ, Duan YY, Liu Q, Xu Q, Jia J. et al. Schiff-base silver nanocomplexes formation on natural biopolymer coated mesoporous silica contributed to the improved curative effect on infectious microbes. *Nano Res* 2021;14:2735–48. 10.1007/s12274-020-3279-6.

[3] Qian X , Lu TL, Huang CQ, Zheng DW, Gong GC, Chu X. et al. Bioinspired sonodynamic nano spray accelerates infected wound healing via targeting and disturbing bacterial metabolism. *Adv Funct Mater* 2024;34:2315576. 10.1002/adfm.202315576.

[4] Cai L , Zhu XY, Ruan HJ, Yang J, Wei W, Wu Y. et al. Curcumin-stabilized silver nanoparticles encapsulated in biocompatible electrospun nanofibrous scaffold for sustained eradication of drug-resistant bacteria. *J Hazard Mater* 2023;452:131290. 10.1016/j.jhazmat.2023.131290. 37023575

[5] Zhu P , Zhou LZ, Song YY, Cai L, Ji MH, Wang J. et al. Encapsulating insoluble antifungal drugs into oleic acid-modified silica mesocomposites with enhanced fungicidal activity. *J Mater Chem B* 2020;8:4899–907. 10.1039/D0TB00106F. 32314756

[6] Xu QL , Zhang L, Liu YH, Cai L, Zhou LZ, Jiang H. et al. Encapsulating MoS_2_-nanoflowers conjugated with chitosan oligosaccharide into electrospun nanofibrous scaffolds for photothermal inactivation of bacteria. *J Nanostructure Chem* 2024;14:137–51. 10.1007/s40097-022-00494-1.

[7] Zhu XY , Wang J, Cai L, Wu Y, Ji MH, Jiang HJ. et al. Dissection of the antibacterial mechanism of zinc oxide nanoparticles with manipulable nanoscale morphologies. *J Hazard Mater* 2022;430:128436. 10.1016/j.jhazmat.2022.128436. 35158241

[8] Sommer R , Wagner S, Rox K, Varrot A, Hauck D, Wamhoff EC. et al. Glycomimetic, orally bioavailable lecb inhibitors block biofilm formation of *Pseudomonas aeruginosa*. *J Am Chem Soc* 2018;140:2537–45. 10.1021/jacs.7b11133. 29272578

[9] Chen MH , Wei JJ, Xie SZ, Tao XY, Zhang ZL, Ran P. et al. Bacterial biofilm destruction by size/surface charge-adaptive micelles. *Nanoscale* 2019;11:1410–22. 10.1039/C8NR05575K. 30608101

[10] Stover CK , Pham XQ, Erwin AL, Mizoguchi SD, Warrener P, Hickey MJ. et al. Complete genome sequence of *Pseudomonas aeruginosa* PAO1, an opportunistic pathogen. *Nature* 2000;406:959–64. 10.1038/35023079. 10984043

[11] Li J , Zhang K, Ruan L, Chin SF, Wickramasinghe N, Liu H. et al. Block copolymer nanoparticles remove biofilms of drug-resistant gram-positive bacteria by nanoscale bacterial debridement. *Nano Lett* 2018;18:4180–7. 10.1021/acs.nanolett.8b01000. 29902011

[12] Durmus NG , Taylor EN, Kummer KM, Webster TJ. Enhanced efficacy of superparamagnetic iron oxide nanoparticles against antibiotic-resistant biofilms in the presence of metabolites. *Adv Mater* 2013;25:5706–13. 10.1002/adma.201302627. 23963848

[13] Flemming HC , Wuertz S. Bacteria and archaea on earth and their abundance in biofilms. *Nat Rev Microbiol* 2019;17:247–60. 10.1038/s41579-019-0158-9. 30760902

[14] Wagner S , Sommer R, Hinsberger S, Lu C, Hartmann RW, Empting M. et al. Novel strategies for the treatment of pseudomonas aeruginosa infections. *J Med Chem* 2016;59:5929–69. 10.1021/acs.jmedchem.5b01698. 26804741

[15] Qin SY , Zhang AQ, Zhang XZ. Recent advances in targeted tumor chemotherapy based on smart nanomedicines. *Small* 2018;14:e1802417. 10.1002/smll.201802417. 30247806

[16] Riley RS , June CH, Langer R, Mitchell MJ. Delivery technologies for cancer immunotherapy. *Nat Rev Drug Discov* 2019;18:175–96. 10.1038/s41573-018-0006-z. 30622344 PMC6410566

[17] Zhang XG , Tang JJ, Li C, Lu Y, Cheng LL, Liu J. A targeting black phosphorus nanoparticle based immune cells nano-regulator for photodynamic/photothermal and photo-immunotherapy. *Bioact Mater* 2021;6:472–89. 10.1016/j.bioactmat.2020.08.024. 32995674 PMC7493086

[18] Yu Z , Chan WK, Zhang Y, Tan TY. Near-infrared-II activated inorganic photothermal nanomedicines. *Biomaterials* 2021;269:120459. 10.1016/j.biomaterials.2020.120459. 33139071

[19] Lian H , Guan P, Tan H, Zhang X, Meng Z. Near-infrared light triggered multi-hit therapeutic nanosystem for tumor specific photothermal effect amplified signal pathway regulation and ferroptosis. *Bioact Mater* 2022;9:63–76. 10.1016/j.bioactmat.2021.07.014. 34820556 PMC8586267

[20] Lan M , Zhao S, Liu W, Lee CS, Zhang W, Wang P. Photosensitizers for photodynamic therapy. *Adv Healthc Mater* 2019;8:1900132. 10.1002/adhm.201900132. 31067008

[21] Luo Y , Li J, Liu X, Tan L, Cui Z, Feng X. et al. Dual metal–organic framework heterointerface. *ACS Cent Sci* 2019;5:1591–601. 10.1021/acscentsci.9b00639. 31572786 PMC6764158

[22] Wei S , Qiao Y, Wu Z, Liu X, Li Y, Cui Z. et al. Na^+^ inserted metal-organic framework for rapid therapy of bacteria-infected osteomyelitis through microwave strengthened Fenton reaction and thermal effects. *Nano Today* 2021;37:101090. 10.1016/j.nantod.2021.101090.

[23] Yuan Z , Lin C, He Y, Tao B, Chen M, Zhang J. et al. Near-infrared light-triggered nitric-oxide-enhanced photodynamic therapy and low-temperature photothermal therapy for biofilm elimination. *ACS Nano* 2020;14:3546–62. 10.1021/acsnano.9b09871. 32069025

[24] Wang Y , Xu Y, Guo X, Wang L, Zeng J, Qiu H. et al. Enhanced antimicrobial activity through the combination of antimicrobial photodynamic therapy and low-frequency ultrasonic irradiation. *Adv Drug Deliv Rev* 2022;183:114168. 10.1016/j.addr.2022.114168. 35189265

[25] Han X , Boix G, Balcerzak M, Moriones OH, Sarabia M, Cortés P. et al. Antibacterial films based on MOF composites that release iodine passively or upon triggering by near-infrared light. *Adv Funct Mater* 2022;32:2112902. 10.1002/adfm.202112902.

[26] Xu M , Ji X, Huo J, Chen J, Liu N, Li Z. et al. Nonreleasing AgNP colloids composite hydrogel with potent hemostatic, photodynamic bactericidal and wound healing-promoting properties. *ACS Appl Mater Interfaces* 2023;15:17742–56. 10.1021/acsami.3c03247. 37006134

[27] Tang Q , Xiao W, Huang C, Si W, Shao J, Huang W. et al. pH-triggered and enhanced simultaneous photodynamic and photothermal therapy guided by photoacoustic and photothermal imaging. *Chem Mater* 2017;29:5216–24. 10.1021/acs.chemmater.7b01075.

[28] Yuan H , Hong X, Ma H, Fu C, Guan Y, Huang W. et al. MXene-based dual functional nanocomposite with photothermal nanozyme catalytic activity to fight bacterial infections. *ACS Mater Lett* 2023;5:762–74. 10.1021/acsmaterialslett.2c00771.

[29] Wang Y , Jin Y, Chen W, Wang J, Chen H, Sun L. et al. Construction of nanomaterials with targeting phototherapy properties to inhibit resistant bacteria and biofilm infections. *Chem Eng J* 2019;358:74–90. 10.1016/j.cej.2018.10.002.

[30] Liu B , Li CX, Chen GY, Liu B, Deng XR, Wei Y. et al. Synthesis and optimization of MoS_2_@Fe_3_O_4_-ICG/Pt (IV) nanoflowers for MR/IR/PA bioimaging and combined PTT/PDT/chemotherapy triggered by 808 nm laser *Adv*. *Sci* 2017;4:1600540. 10.1002/advs.201600540 PMC5566229 28852616

[31] Younis MR , Wang C, An R, Wang S, Younis MA, Li ZQ. et al. Low power single laser activated synergistic cancer phototherapy using photosensitizer functionalized dual plasmonic photothermal nanoagents. *ACS Nano* 2019;13:2544–57. 10.1021/acsnano.8b09552. 30730695

[32] Liu Y , Guo Z, Li F, Xiao Y, Zhang Y, Bu T. et al. Multifunctional magnetic copper ferrite nanoparticles as Fenton-like reaction and near-infrared photothermal agents for synergetic antibacterial therapy. *ACS Appl Mater Interfaces* 2019;11:31649–60. 10.1021/acsami.9b10096. 31407880

[33] Zhang W , Hu S, Yin JJ, He W, Lu W, Ma M. et al. Prussian blue nanoparticles as multienzyme mimetics and reactive oxygen species scavengers. *J Am Chem Soc* 2016;138:5860–5. 10.1021/jacs.5b12070. 26918394

[34] Li J , Liu X, Tan L, Cui Z, Yang X, Liang Y. et al. Zinc-doped Prussian blue enhances photothermal clearance of *Staphylococcus aureus* and promotes tissue repair in infected wounds. *Nat Commun* 2019;10:4490. 10.1038/s41467-019-12429-6. 31582736 PMC6776522

[35] Andrews SC , Robinson AK, Quiñones F. Bacterial iron homeostasis. *FEMS Microbiol Rev* 2003;27:215–37. 10.1016/S0168-6445(03)00055-X. 12829269

[36] Hu R , Fang Y, Huo M, Yao H, Wang C, Chen Y. et al. Ultrasmall Cu_2_-xS nanodots as photothermal-enhanced Fenton nanocatalysts for synergistic tumor therapy at NIR-II biowindow. *Biomaterials* 2019;206:101–14. 10.1016/j.biomaterials.2019.03.014. 30927714

[37] Xie X , Wang R, Zhang X, Ren Y, Du T, Ni Y. et al. A photothermal and self-induced Fenton dual-modal antibacterial platform for synergistic enhanced bacterial elimination. *Appl Catal B Environ* 2021;295:120315. 10.1016/j.apcatb.2021.120315.

[38] Zhou F , Lin S, Zhang J, Kong Z, Tan BK, Hamzah SS. et al. Enhancement of photodynamic bactericidal activity of curcumin against *Pseudomonas aeruginosa* using polymyxin B. *Photodiagn Photodyn Ther* 2022;37:102677. 10.1016/j.pdpdt.2021.102677. 34890782

[39] Wu W , Yu L, Pu Y, Yao H, Chen Y, Shi J. Copper-enriched prussian blue nanomedicine for in situ disulfiram toxification and photothermal antitumor amplification. *Adv Mater* 2020;32:e2000542. 10.1002/adma.202000542. 32162734

[40] Su CH , Li WP, Tsao LC, Wang LC, Hsu YP, Wang WJ. et al. Enhancing microcirculation on multitriggering manner facilitates angiogenesis and collagen deposition on wound healing by photoreleased NO from hemin-derivatized colloids. *ACS Nano* 2019;13:4290–301. 10.1021/acsnano.8b09417. 30883107

[41] Ren B , Jones LA, Oppedisano DK, Kandjani AE, Chen M, Antolasic F. et al. The preparation of a AuCN/Prussian blue nanocube composite through galvanic replacement enhances stability for electrocatalysis. *Chemistry Select* 2017;2:5333–40. 10.1002/slct.201700908.

[42] Wang C , Wang Y, Xu L, Zhang D, Liu M, Li X. et al. Facile aqueous-phase synthesis of biocompatible and fluorescent Ag_2_S nanoclusters for bioimaging: tunable photoluminescence from red to near infrared. *Small* 2012;8:3137–42. 10.1002/smll.201200376. 22777739

[43] Wang Y , Zhao Y, Wu J, Li M, Tan J, Fu W. et al. Negatively charged sulfur quantum dots for treatment of drug-resistant pathogenic bacterial infections. *Nano Lett* 2021;21:9433–41. 10.1021/acs.nanolett.1c02697. 34752115

[44] Li J , Zhang Q, Chen Z, Guo S, Guo J, Yan F. Postsynthetic modification of thermo-treated metal–organic framework for combined photothermal/photodynamic antibacterial therapy. *ACS Appl Mater Interfaces* 2024;16:8459–73. 10.1021/acsami.3c17955. 38327180

[45] Ci D , Wang N, Xu Y, Wu S, Wang J, Li H. et al. SiO_2_@AuAg/PDA hybrid nanospheres with photo-thermally enhanced synergistic antibacterial and catalytic activity. *RSC Adv* 2024;14:4518–32. 10.1039/D3RA07607E. 38312727 PMC10836413

[46] Wang C , Chen Q, Yin R, Yuan X, Kang H, Cai A. et al. Photothermal effect and antimicrobial properties of cerium-doped bioactive glasses. *Ceram Int* 2024;50:20235–46. 10.1016/j.ceramint.2024.03.147.

[47] Xie M , Gao M, Yun Y, Malmsten M, Rotello VM, Zboril R. et al. Antibacterial nanomaterials: mechanisms, impacts on antimicrobial resistance and design principles. *Angew Chem Int Ed* 2023;62:e202217345. 10.1002/anie.202217345. 36718001

[48] Miller MB , Bassler BL. Quorum sensing in bacteria. *Ann Rev Microbiol* 2001;55:165–99. 10.1146/annurev.micro.55.1.165. 11544353

[49] Welsh MA , Eibergen NR, Moore JD, Blackwell HE. Small molecule disruption of quorum sensing cross-regulation in *Pseudomonas aeruginosa* causes major and unexpected alterations to virulence phenotypes. *J Am Chem Soc* 2015;137:1510–9. 10.1021/ja5110798. 25574853 PMC4372995

[50] Kong KF , Jayawardena SR, Indulkar SD, del Puerto A, Koh CL, Høiby N. et al. *Pseudomonas aeruginosa* AmpR is a global transcriptional factor that regulates expression of AmpC and PoxB β-lactamases, proteases, quorum sensing, and other virulence factors. *Antimicrob Agents Chemother* 2005;49:4567–75. 10.1128/AAC.49.11.4567-4575.2005. 16251297 PMC1280116

[51] Li Z , You S, Mao R, Xiang Y, Cai E, Deng H. et al. Architecting polyelectrolyte hydrogels with Cu-assisted polydopamine nanoparticles for photothermal antibacterial therapy. *Mater Today Bio* 2022;15:100264. 10.1016/j.mtbio.2022.100264. PMC9062430 35517578

[52] Yin M , Wu J, Deng M, Wang P, Ji G, Wang M. et al. Multifunctional magnesium organic framework-based microneedle patch for accelerating diabetic wound healing. *ACS Nano* 2021;15:17842–53. 10.1021/acsnano.1c06036. 34761898

[53] He X , Dai L, Ye L, Sun X, Enoch O, Hu R. et al. A vehicle-free antimicrobial polymer hybrid gold nanoparticle as synergistically therapeutic platforms for *Staphylococcus aureus* infected wound healing. *Adv Sci* 2022;9:2105223. 10.1002/advs.202105223. PMC9108595 35274475

[54] Deng Z , Shi F, Zhou Z, Sun F, Sun MH, Sun Q. et al. M1 macrophage mediated increased reactive oxygen species (ROS) influence wound healing via the MAPK signaling *in vitro* and *in vivo*. *Toxicol Appl Pharmacol* 2019;366:83–95. 10.1016/j.taap.2019.01.022. 30690042

[55] Murray PJ , Wynn TA. Protective and pathogenic functions of macrophage subsets. *Nat Rev Immunol* 2011;11:723–37. 10.1038/nri3073. 21997792 PMC3422549

[56] Hernando S , Coque TM, Baquero F, Martínez JL. Defining and combating antibiotic resistance from one health and global health perspectives. *Nat Microbiol* 2019;4:1432–42. 10.1038/s41564-019-0503-9. 31439928

[57] Reyes J , Komarow L, Chen L, Ge L, Hanson BM, Cober E. et al. Global epidemiology and clinical outcomes of carbapenem-resistant Pseudomonas aeruginosa and associated carbapenemases (POP): a prospective cohort study. *Lancet Microbe* 2023;4:e159–70. 10.1016/S2666-5247(22)00329-9. 36774938 PMC10016089

[58] Huang T , He X, Ali A, Gnanasekar S, Xiang Y, Zhang K. et al. Phytic acid-promoted deposition of gold nanoparticles with grafted cationic polymer brushes for the construction of synergistic contact-killing and photothermal bactericidal coatings. *ACS Appl Bio Mater* 2024;7:3283–94. 10.1021/acsabm.4c00237. 38727030

[59] Tian H , Zhu H, Xue Y, Wang M, Xing K, Li Z. et al. White light powered antimicrobial nanoagents for triple photothermal, chemodynamic and photodynamic based sterilization. *Nanoscale Horiz* 2024;9:659–874. 10.1039/D4NH00060A. 38757185

[60] Li J , Pan G, Zyryanov GV, Peng Y, Zhang G, Ma L. et al. Positively charged semiconductor conjugated polymer nanomaterials with photothermal activity for antibacterial and antibiofilm activities *in vitro* and *in vivo*. *ACS Appl Mater Interfaces* 2023;15:40864–76. 10.1021/acsami.3c00556. 37603418

[61] Zhong J , Xiao F, Jin B, Zhang G, Hou H, Bi J. et al. Construction of sandwich-structured V_2_CTx@PDA@Ag nanosheet composite for enhanced near-infrared photothermocatalytic sterilization performance. *Mater Chem Phys* 2024;314:128931. 10.1016/j.matchemphys.2024.128931.

[62] Xu B , Yu D, Xu C, Gao Y, Sun H, Liu L. et al. Study on synergistic mechanism of molybdenum disulfide/sodium carboxymethyl cellulose composite nanofiber mats for photothermal/photodynamic antibacterial treatment. *Int J Biol Macromol* 2024;266:130838. 10.1016/j.ijbiomac.2024.130838. 38521322

[63] Li X , Zhou J, Dong X, Cheng WY, Duan H, Cheung PCK. In Vitro and In Vivo Photothermal cancer therapeutic effects of gold nanorods modified with mushroom β-Glucan. J Agric Food Chem. 2018;66 4091–8. 10.1021/acs.jafc.8b00292. 29627979

