# Supplementary material for: Thermo-responsive cascade antimicrobial platform for precise biofilm removal and enhanced wound healing
Source: Burns Trauma. 2024 Sep 25;12:tkae038. doi: 10.1093/burnst/tkae038 (PMC11422504; doi:10.1093/burnst/tkae038)
Supplement: Supplementary_material_tkae038 [file supplementary_material_tkae038.zip › Figure S1.docx]

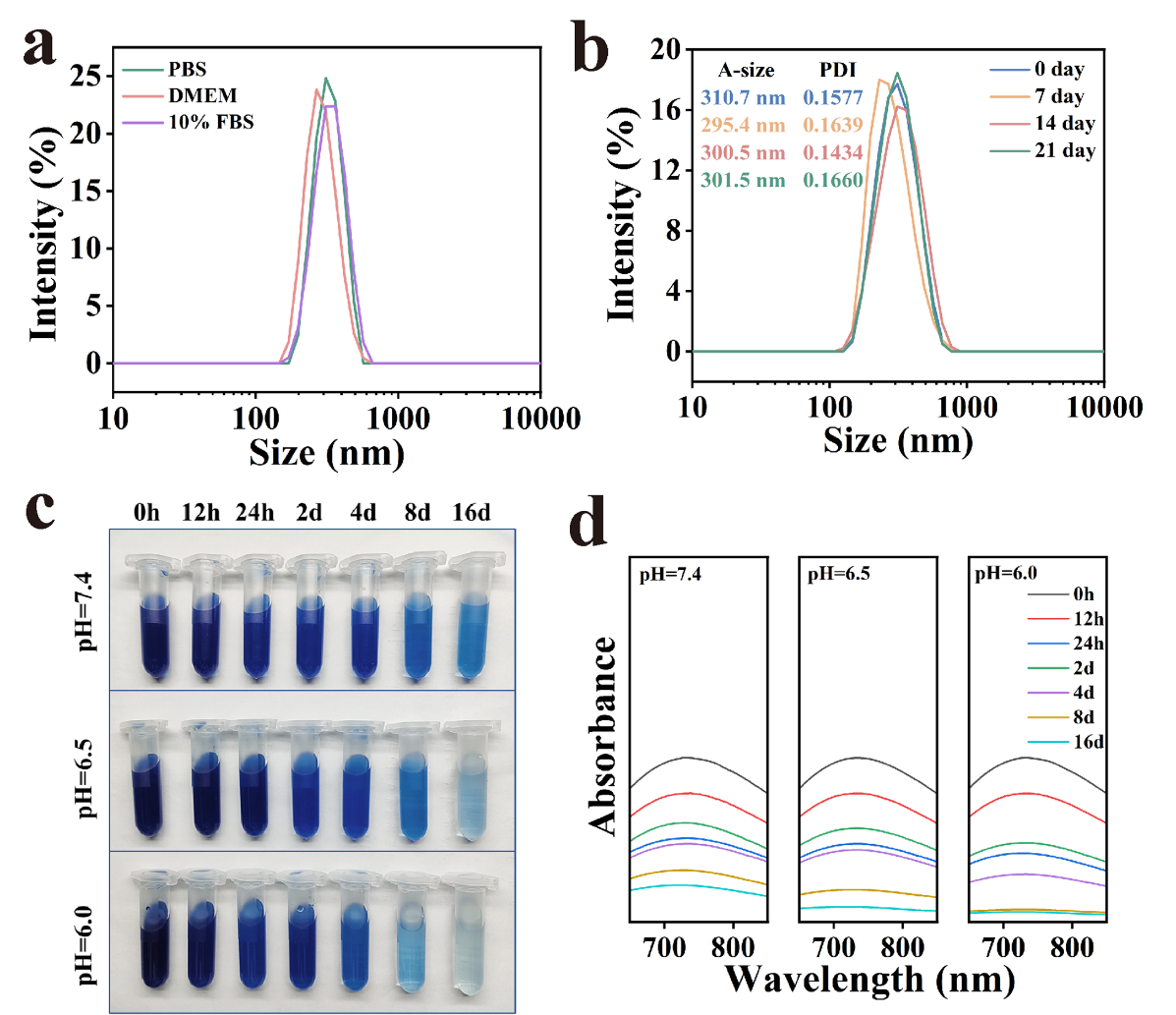


**Figure S1.** Biodegradability of HMAPH. (a) Hydrodynamic sizes of HMAPH in different solutions, including PBS, DMEM cell medium and serum. (b) Hydrodynamic sizes of HMAPH over time in PBS. (c) Photographs of Eppendorf tubes containing HMAPH solutions of different pH values after oscillation at different durations. (d) UV-vis spectra of HMAPH immersed in PBS with different pH values at a different time period. *HMPB* hollow mesoporous prussian blue, *MB* methylene blue, *PMB* polymyxin b, *HA* hyaluronic acid， *HMAPH* HMPB@MB@AuNPs@PMB@HA, *PBS* phosphate-buffered saline, *UV-Vis* ultraviolet–visible spectroscopy.
