# Supplementary material for: Thermo-responsive cascade antimicrobial platform for precise biofilm removal and enhanced wound healing
Source: Burns Trauma. 2024 Sep 25;12:tkae038. doi: 10.1093/burnst/tkae038 (PMC11422504; doi:10.1093/burnst/tkae038)
Supplement: Supplementary_material_tkae038 [file supplementary_material_tkae038.zip › Figure S10.docx]

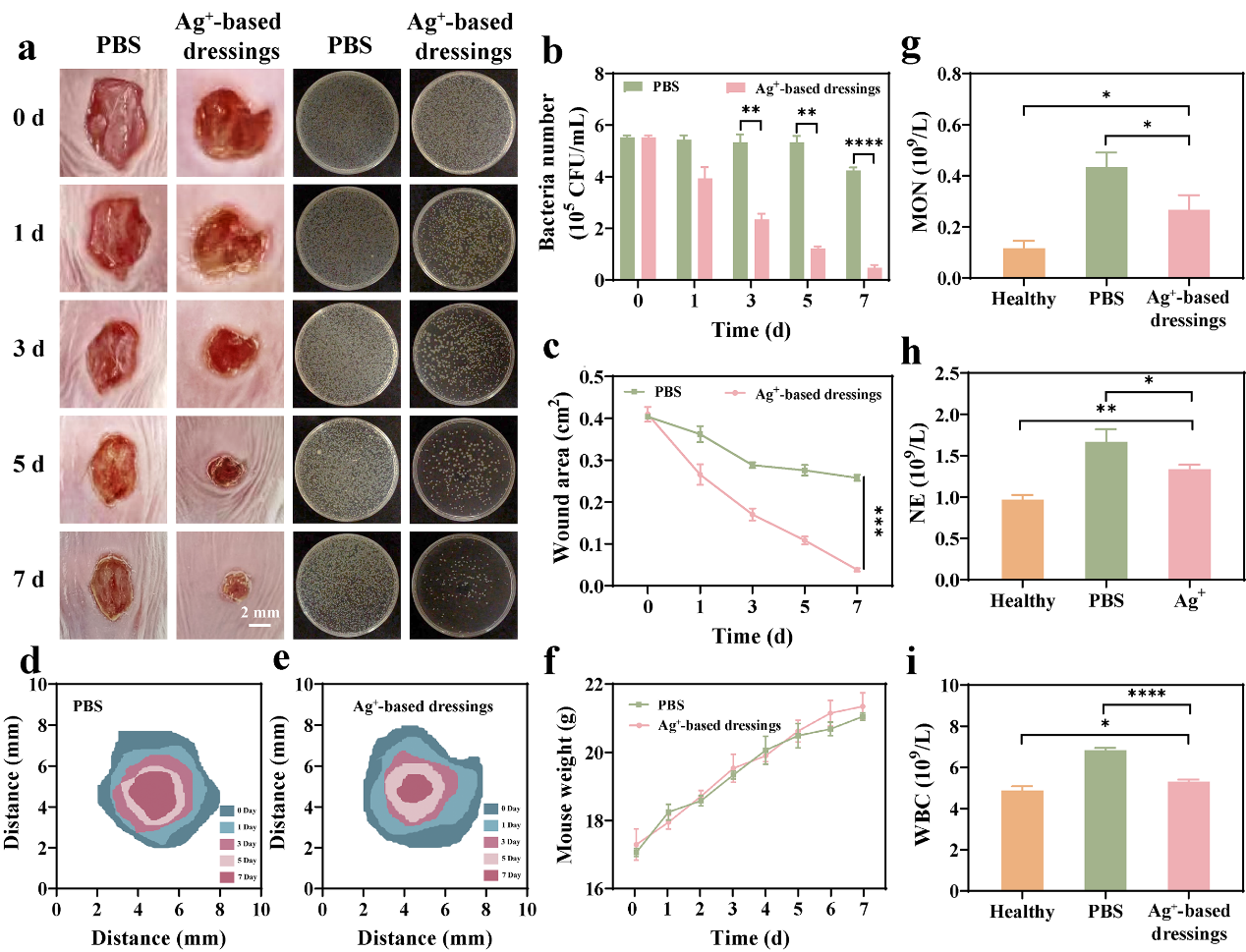


**Figure S10.** *In vivo* evaluation of silver-containing dressings for *P. aeruginosa*-infected wounds. (a) Thermal images of abscesses in mice treated with silver-containing dressings and photographs of mice wounds after 7 days of various treatments. (b) Quantitative measurement of wound bacterial counts of treatment in different groups. (c) Statistical chart of mice wound area in different treatment groups at different treatment time points (day). (d-e) Dynamic wound healing process within 7 days in different treatment group groups. (f) Weight changes in different treatment groups over different treatment periods. Changes of monocyte (MONO) (g), neutrophil (NEUT) (h), white blood cell (WBC) (i), in *P. aeruginosa*-infected mice after 7 days of two different treatments. *PBS* phosphate-buffered saline.
