# Supplementary material for: Thermo-responsive cascade antimicrobial platform for precise biofilm removal and enhanced wound healing
Source: Burns Trauma. 2024 Sep 25;12:tkae038. doi: 10.1093/burnst/tkae038 (PMC11422504; doi:10.1093/burnst/tkae038)
Supplement: Supplementary_material_tkae038 [file supplementary_material_tkae038.zip › Figure S11.docx]

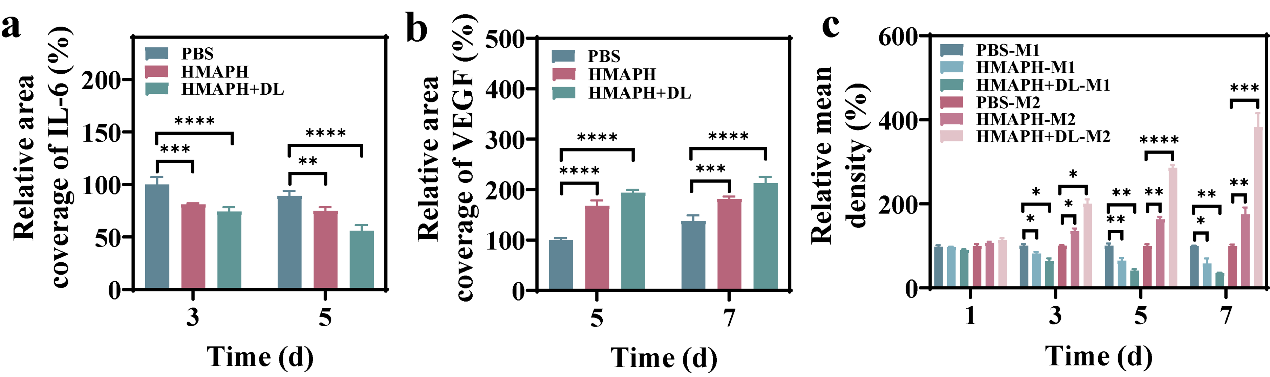


**Figure S11**. Fluorescence intensity corresponding to different immune factors. Quantified data of the relative area coverage of IL-6 (a) and VEGF (b). (c) Quantitative data of M1 and M2 immunofluorescence cumulative optical density values on day 7 in different treatment groups. *DL* dual light, *PBS* phosphate-buffered saline.
