# Supplementary material for: Thermo-responsive cascade antimicrobial platform for precise biofilm removal and enhanced wound healing
Source: Burns Trauma. 2024 Sep 25;12:tkae038. doi: 10.1093/burnst/tkae038 (PMC11422504; doi:10.1093/burnst/tkae038)
Supplement: Supplementary_material_tkae038 [file supplementary_material_tkae038.zip › Figure S12.docx]

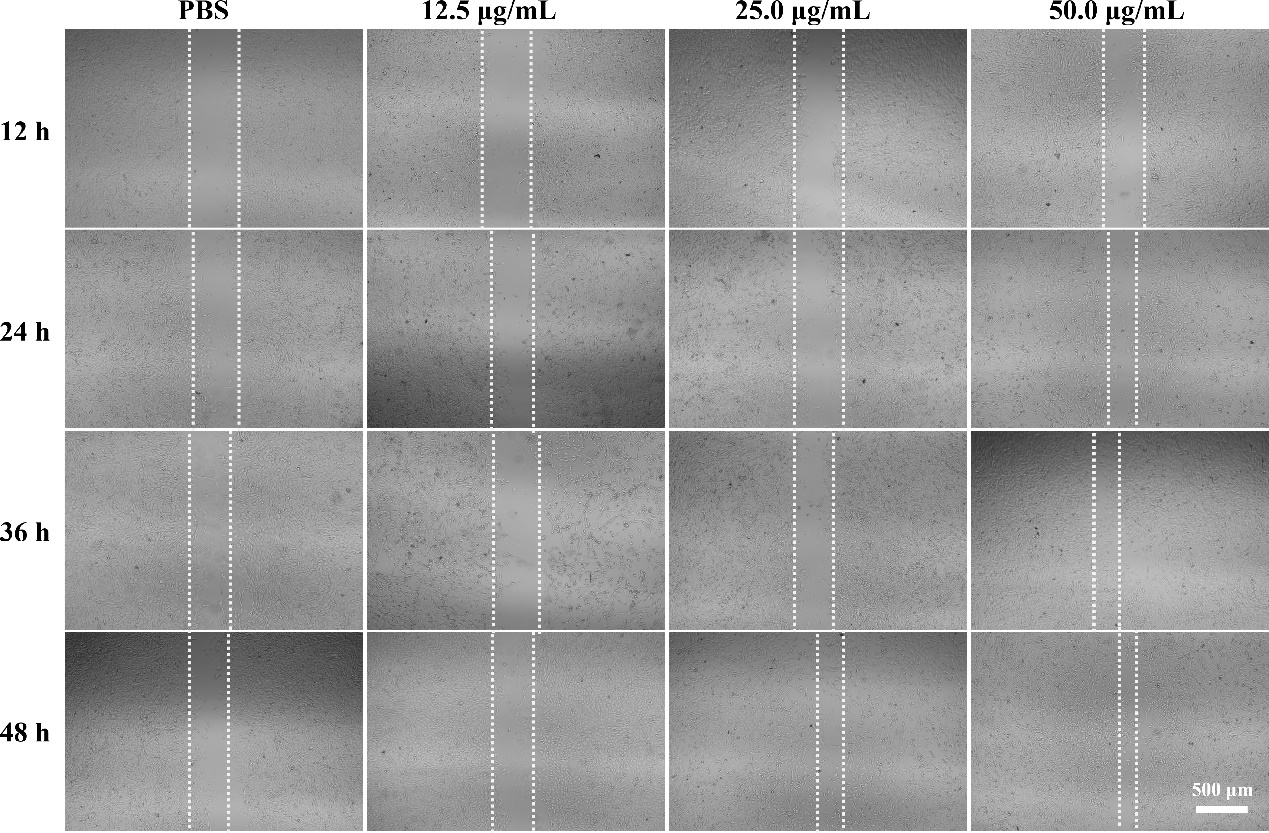


**Figure S12.** Fibroblast migration experiments with different concentrations of HMAPH at different incubation time intervals. *PBS* phosphate-buffered saline.
