# Supplementary material for: Thermo-responsive cascade antimicrobial platform for precise biofilm removal and enhanced wound healing
Source: Burns Trauma. 2024 Sep 25;12:tkae038. doi: 10.1093/burnst/tkae038 (PMC11422504; doi:10.1093/burnst/tkae038)
Supplement: Supplementary_material_tkae038 [file supplementary_material_tkae038.zip › Figure S2.docx]

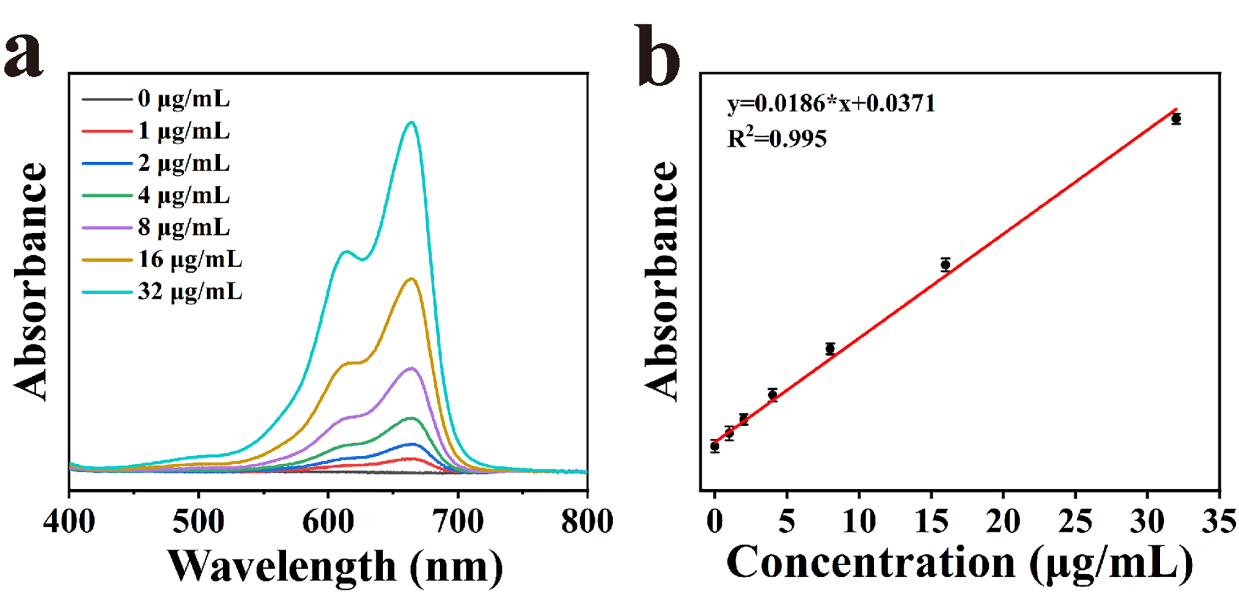


**Figure S2.** Standard concentration curves for MB. (a) The UV-vis absorption of MB at different concentrations. (b) The linear relationship between MB concentration and absorption intensity. *HMPB* hollow mesoporous prussian blue, *MB* methylene blue, *PMB* polymyxin b, *HA* hyaluronic acid， *HMAPH* HMPB@MB@AuNPs@PMB@HA, *UV-Vis* ultraviolet–visible spectroscopy.
