# Supplementary material for: Thermo-responsive cascade antimicrobial platform for precise biofilm removal and enhanced wound healing
Source: Burns Trauma. 2024 Sep 25;12:tkae038. doi: 10.1093/burnst/tkae038 (PMC11422504; doi:10.1093/burnst/tkae038)
Supplement: Supplementary_material_tkae038 [file supplementary_material_tkae038.zip › Figure S3.docx]

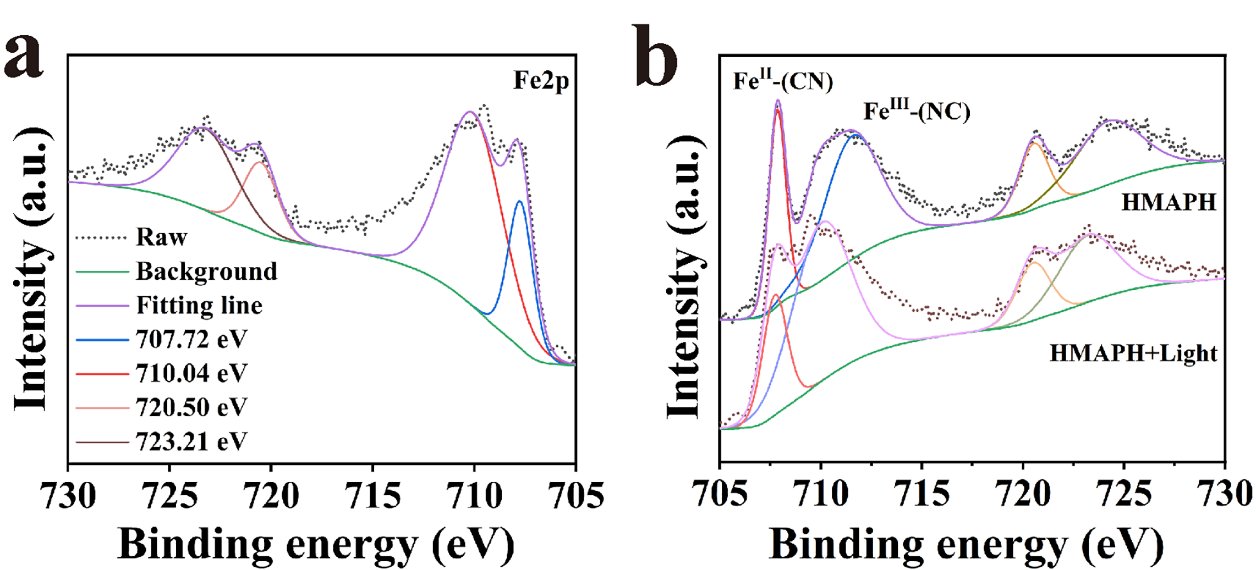


**Figure S3.** XPS of HMAPH before and after NIR light irradiation. (a) XPS spectra of Fe2p of HMAPH after NIR light irradiation. (b) XPS spectra of Fe II-(CN) and Fe III-(NC) after NIR light irradiation. *NIR* near infrared, *XPS* x-ray photoelectron spectroscopy.
