# Supplementary material for: Thermo-responsive cascade antimicrobial platform for precise biofilm removal and enhanced wound healing
Source: Burns Trauma. 2024 Sep 25;12:tkae038. doi: 10.1093/burnst/tkae038 (PMC11422504; doi:10.1093/burnst/tkae038)
Supplement: Supplementary_material_tkae038 [file supplementary_material_tkae038.zip › Figure S4.docx]

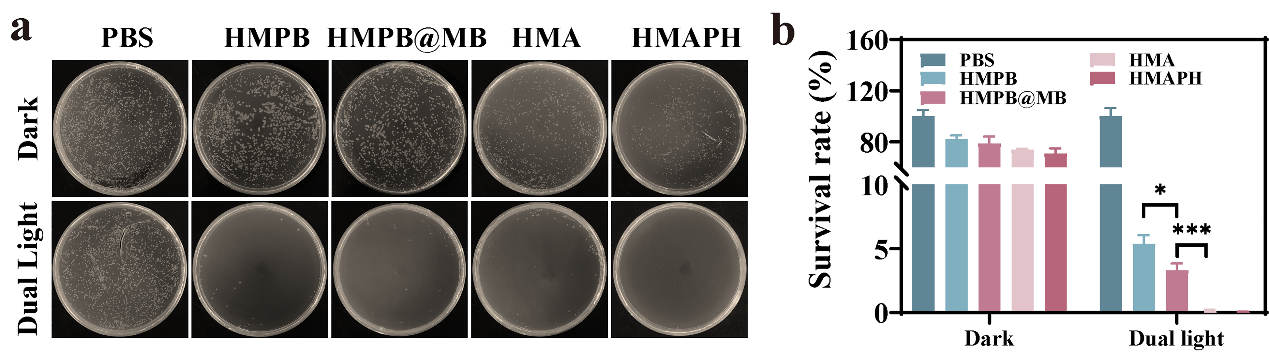


**Figure S4.** Antimicrobial properties of nanoparticles with different synthesis steps. Photographs of colonies formed by *P. aeruginosa* treated with different materials (HMPB, HMPB@MB, HMA, HMAPH) at the same concentration (50.0 μg/mL) under dark conditions and dual light irradiation for 10 min (a) and the corresponding antibacterial rates of *P. aeruginosa* (b). *p <0.05, ***p < 0.001. *MB* methylene blue, *HA* hyaluronic acid，*PMB* polymyxin b, *HMPB* hollow mesoporous prussian blue, *HMA* HMPB@MB@AuNPs, *HMAP* HMPB@MB@AuNPs@PMB, *HMAPH* HMPB@MB@AuNPs@PMB@HA, *PBS* phosphate-buffered saline.
