# Supplementary material for: Thermo-responsive cascade antimicrobial platform for precise biofilm removal and enhanced wound healing
Source: Burns Trauma. 2024 Sep 25;12:tkae038. doi: 10.1093/burnst/tkae038 (PMC11422504; doi:10.1093/burnst/tkae038)
Supplement: Supplementary_material_tkae038 [file supplementary_material_tkae038.zip › Figure S5.docx]

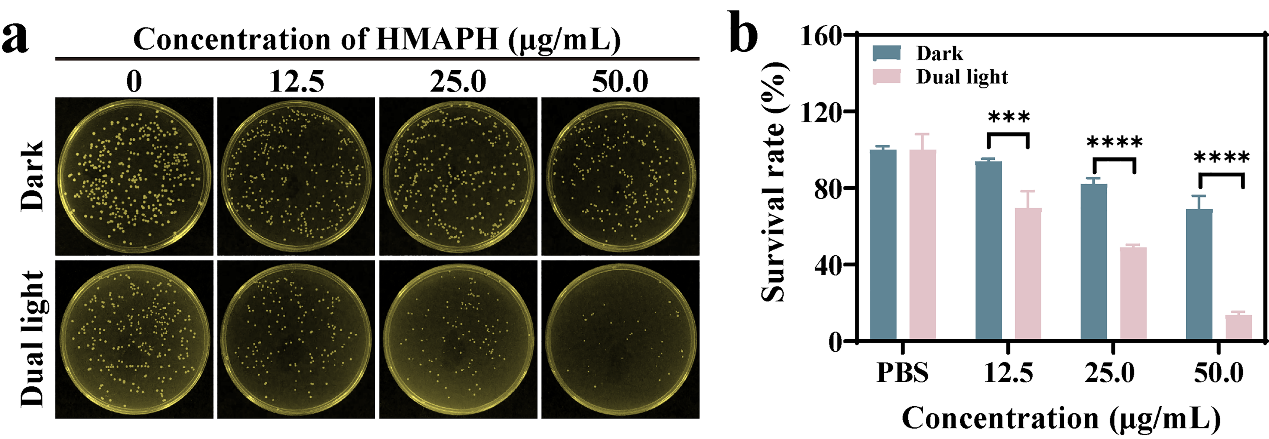


**Figure S5.** HMAPH antimicrobial properties against *S. aureus*. The relative bacterial viability of *S. aureus* (a). Photographs of bacterial colonies formed by *S. aureus* (b) after exposure to different concentrations of HMAPH with or without NIR irradiation. ****P* *<* 0.001 and *****P* *<* 0.0001. *MB* methylene blue, *HA* hyaluronic acid，*PMB* polymyxin b, *HMPB* hollow mesoporous prussian blue, *HMA* HMPB@MB@AuNPs, *HMAP* HMPB@MB@AuNPs@PMB, *HMAPH* HMPB@MB@AuNPs@PMB@HA, *PBS* phosphate-buffered saline, *S. aureus Staphylococcus aureus*.
