# Supplementary material for: Thermo-responsive cascade antimicrobial platform for precise biofilm removal and enhanced wound healing
Source: Burns Trauma. 2024 Sep 25;12:tkae038. doi: 10.1093/burnst/tkae038 (PMC11422504; doi:10.1093/burnst/tkae038)
Supplement: Supplementary_material_tkae038 [file supplementary_material_tkae038.zip › Figure S6.docx]

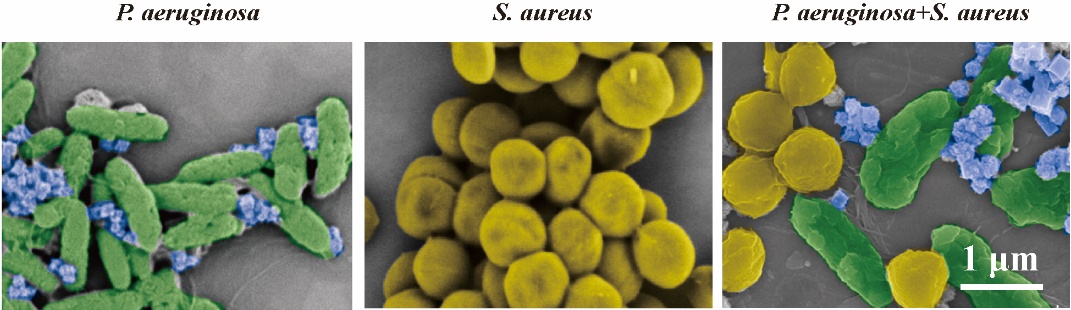


**Figure S6.** SEM images of *P. aeruginosa, S. aureus*, *S. aureus* and *P. aeruginosa* mixed bacterial suspension treated with HMAPH at 50.0 μg/mL. *MB* methylene blue, *HA* hyaluronic acid，*PMB* polymyxin b, *HMPB* hollow mesoporous prussian blue, *HMA* HMPB@MB@AuNPs, *HMAP* HMPB@MB@AuNPs@PMB, *HMAPH* HMPB@MB@AuNPs@PMB@HA, *PBS* phosphate-buffered saline, *S. aureus Staphylococcus aureus*, *P. aeruginosa* *Pseudomonas aerginosa*, *SEM* scanning electron microscope.
