# Supplementary material for: Thermo-responsive cascade antimicrobial platform for precise biofilm removal and enhanced wound healing
Source: Burns Trauma. 2024 Sep 25;12:tkae038. doi: 10.1093/burnst/tkae038 (PMC11422504; doi:10.1093/burnst/tkae038)
Supplement: Supplementary_material_tkae038 [file supplementary_material_tkae038.zip › Figure S9.docx]

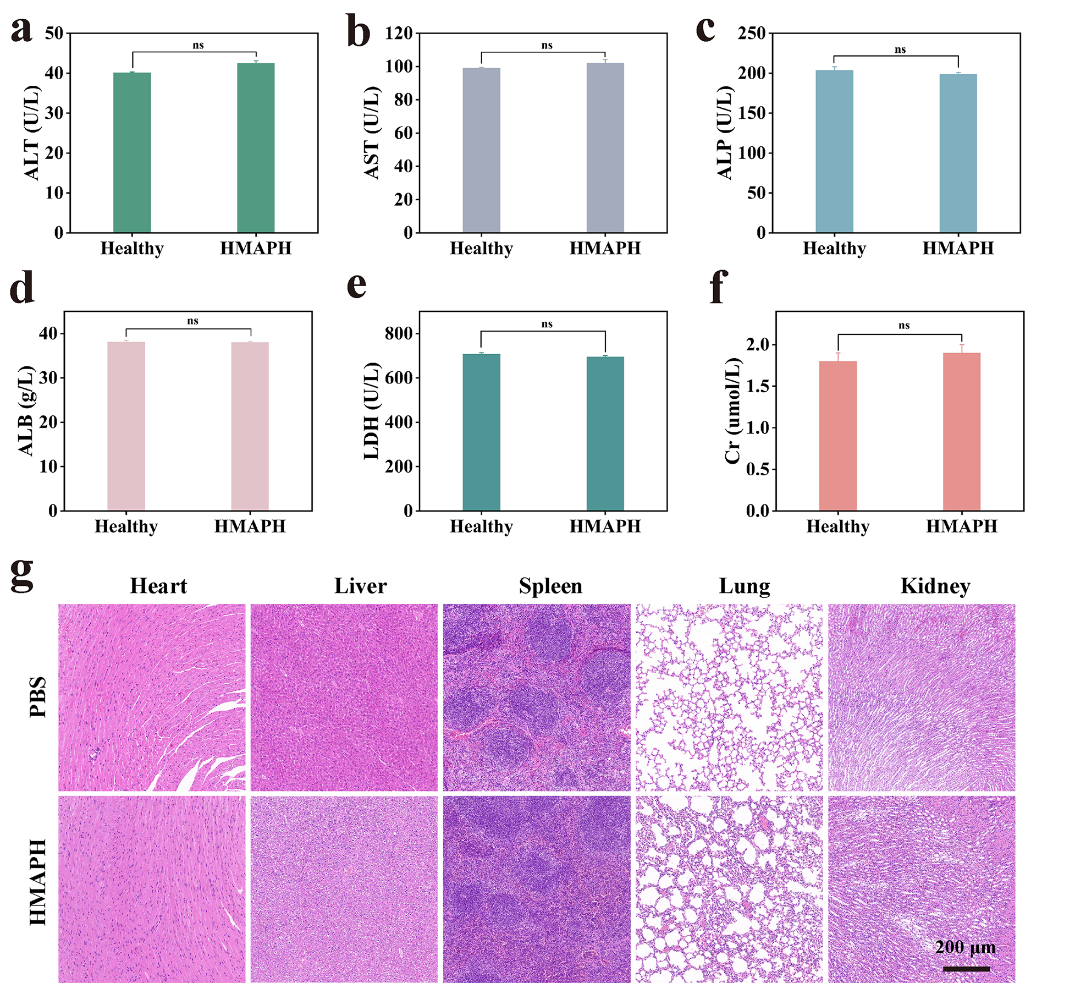


**Figure S9.** Acute toxicity assay of HMAPH *in vivo*. Changes of major serum biochemistry indicators of alanine transaminase (ALT) (a), aspartate transaminase (AST) (b), alkaline phosphatase (ALP) (c), albumin (ALB) (d), lactate dehydrogenase (LDH) (e) and creatinine (Cr) (f) in normal mice after treatment with PBS and HMAPH (200 μL, 50.0 μg/mL). (g) H&E staining of internal organs (heart, liver, spleen, lung and kidney) after treatment with PBS and HMAPH. ns: not statistically different at *p*<0.05. *H&E* hematoxylin & eosin, *PBS* phosphate-buffered saline.
