# Supplementary material for: Thermo-responsive cascade antimicrobial platform for precise biofilm removal and enhanced wound healing
Source: Burns Trauma. 2024 Sep 25;12:tkae038. doi: 10.1093/burnst/tkae038 (PMC11422504; doi:10.1093/burnst/tkae038)
Supplement: Supplementary_material_tkae038 [file supplementary_material_tkae038.zip › Supporting_information_tkae038(1).docx]

Thermo-responsive cascade antimicrobial platform for precise biofilm removal and enhanced wound healing

**1.** **Experimental**

**1.1. Materials**

Polyvinyl pyrrolidone (PVP, K30) was obtained from Solarbio Sci-Tech Co. (Beijing, China); Polymyxin B sulfate (≥6000 USP units/mg) (PMB) and potassium ferricyanide (K3[Fe(CN)6]) from Aladdin (Shanghai, China); Hyaluronic acid (HA, WM: 10-20 KDa) from Macklin Reagent Co. (Shanghai, China); COOH-PEG-SH (WM: 2000 Da) and N-Hydroxysuccinimide (NHS) from Shanghai Yuanye Bio-technology Co., Ltd. (Shanghai, China); 1-(3-Dimethylaminopropyl)-3-ethylcarbodiimide hydrochloride (EDC) from Beyotime (Beijing, China); Glutathione (GSH) from Jiancheng Biotechnology Co. (Nanjing, China); Rhodamine B (RhB) from Merck KGaA. (USA). The strains of *Pseudomonas aeruginosa* (*P. aeruginosa*, CICC 35150) used in this study were purchased from China center of industrial culture collection (Beijing, China).

**1.2. Photothermal effect of HMAPH**

Temperature changes of different concentrations of HMAPH solution (50.0 μg/mL, 25.0 μg/mL and 12.5 μg/mL) were recorded under 808 nm NIR laser irradiation (1.0 W/cm^2^, BST808-4000-F). Real-time temperature and thermal images of different concentrations of HMAPH under NIR irradiation for 10 min were monitored using a FLUKE Ti400+ thermal imaging camera. The thermal stability of HMAPH was investigated by NIR irradiation for 10 min (808 nm, 1.0 W/cm^2^) followed by natural cooling to room temperature for 10 consecutive on-off cycles. on efficiency of HMAPH was investigated using Yang’s method [1].

**1.3. Fenton reaction**

Briefly, 1×10^−3^ M glutathione (GSH) was mixed with HMAPH (50.0 μg/mL), followed by adding Rhodamine B (RhB, 1×10^−6^ M) and H_2_O_2_ (1×10^−6^ M) for 2 h of adsorption/desorption. Finally, the absorbance of mixture at 500-600 nm was measured with a microplate reader.

**1.4. *In vitro* antibacterial activity**

*P. aeruginosa* (10^6^ CFU/mL) was treated separately with different concentrations of HMAPH (50.0 μg/mL, 25.0 μg/mL, 12.5 μg/mL) for 10 min under dual light laser irradiation or dark conditions, using PBS-treated bacteria as a blank control. Next, 50 μL of each treated bacterial suspension was coated on the LB agar medium and incubated at 37 °C overnight. The bacterial survival rate was calculated by the equation: survival rate (%) = CFU_blank group_/CFU_experimental group_ × 100%.

**1.5. Bacterial morphology observations**

After various treatments, *P. aeruginosa* morphology was analyzed by transmission electron microscopy (TEM) and scanning electron microscopy (SEM). Briefly, bacterial suspensions treated with HMAPH (50.0 μg/mL) were collected in the presence or absence of dual light laser irradiation, followed by fixation with 100 μL of 2.5% glutaraldehyde overnight. Next, the bacteria were dehydrated with different gradients of ethanol for 10 min and resuspended in 100% ethanol. Finally, the suspension droplets were placed on ultrathin carbon grids or glass slides for TEM (Talos F200X G2, FEI Czechoslovakia Ltd. The Czech Republic) and SEM (apero, FEI Czechoslovakia Ltd. USA) observation, respectively.

**1.6. Live/dead staining assay and extracellular ROS detection**

The bacterial suspensions in various treatment groups were collected and washed with PBS, followed by staining with PI (10 µg/mL) for 15 min and DAPI (5 µg/mL) for 5 min in the dark. An inverted fluorescence microscope (AMG evos) was used to observe the fluorescence of live and dead bacteria.

After various treatments, bacteria were stained with DCFH-DA (10 μM) to assess changes in bacterial ROS levels. After several washes with PBS, the fluorescence intensity of the bacteria was observed with an AMG evos inverted fluorescence microscope (Fisher Scientific, Waltham, MA, USA).

**1.7. Detection of protein leakage and genomic DNA**

*P. aeruginosa* (10^6^ CFU/mL) was treated separately with PBS and HMAPH (50.0 μg/mL) in the presence or absence of dual light laser irradiation for 10 min. After centrifugation to remove the supernatant, the protein content of each treatment group was determined by the BCA protein concentration assay kit as instructed by the manufacturer (cat#PC0020, Solarbio, China).

Bacterial genomic DNA was extracted from the above differently treated bacterial suspensions using the Genomic DNA Extraction Kit as instructed by the manufacturer (TIANGEN BIOTECH, Beijing). The concentration of DNA was quantified by measuring the absorbance at 260 nm with a microplate reader (TITERTEK ERTHOLD, Germany).

**1.8. Detection of bacterial Atpase and catase activity**

*P. aeruginosa* (10^6^ CFU/mL) was treated separately with PBS and HMAPH (50.0 μg/mL) in the presence or absence of dual light laser irradiation for 10 min, followed by centrifugation and washing with PBS to obtain the cell pellets. Next, the cell pellets of each group were resuspended in 4.0 mL PBS and then placed in a high-pressure cell disruptor for cell wall-breaking treatment. Finally, the levels of ATPase activity and catalase (CAT) activity of each group were measured by ATPase test kit and CATase assay kit as instructed by the manufacturer (Jiancheng Biotechnology Co., Nanjing, China).

**1.9. *In vitro* cytotoxicity assay**

The *in vitro* cytotoxicity of HMAPH was investigated by MTT assay. Briefly, after seeding into 96-well plates, the NIH-3T3 cells were incubated for 24 h until they grew into monolayer, followed by discarding the supernatant, adding different concentrations of HMAPH (50.0 μg/mL, 25.0 μg/mL and 12.5 μg/mL), and incubation for 12 and 24 h, respectively. Next, 20 μL of MTT (5.0 mg/mL) was injected into each well, followed by incubation for 4 h and then adding 150 μL/well of DMSO. Finally, the absorbance of each well was measured at 490 nm by a microplate reader and cell viability was calculated (Molecular Devices SpectraMax i3x, Molecular Devices, USA).

**1.10. Hemolysis assay**

The *in vitro* hemolysis activity of HMAPH was evaluated by BALB/c mice’s fresh red blood cells (RBCs). Briefly, the suspensions of HMAPH (in PBS, 2.0 mL) at different concentrations (50.0 μg/mL, 25.0 μg/mL and 12.5 μg/mL) were gently mixed with 25 μL RBCs and incubated for 30 min at 37 °C in a water bath. After centrifugation, the absorbance of the supernatant at 545 nm was measured using a multi-plate reader (Molecular Devices SpectraMax i3x, Molecular Devices, USA). Meanwhile, PBS and deionized water were added as negative and positive control group, respectively. The hemolysis rate was estimated by the equation: Hemolysis rate% = (sample absorption value negative absorption value)/(positive absorption value - negative absorption value) ×100%.

**1.11. Biofilm formation and evaluation of biofilm inhibition *in vitro***

Briefly, 100 μL of *P. aeruginosa* (OD = 0.6-0.8) was placed in a 96-well plate and cultured in a 37°C incubator to form a biofilm. After culturing for 48 h, the biofilms were treated separately with PBS, PBS+dual light, HMAPH and HMAPH+dual light. After 10 min of dual laser irradiation or no irradiation, the supernatant was discarded, followed by adding 200 μL of 99% methanol, incubation for 15 min to fix the biofilm, and then adding 0.1% crystal violet. After 30 min of binding, the supernatant was discarded and the biofilms were washed 3 times with sterile water. Finally, 100 μL of 95% ethanol was added to release the bound crystal violet. The absorbance of each treatment group was measured at 570 nm using a multifunctional microplate reader. (Molecular Devices SpectraMax i3x, Molecular Devices, USA).

Furthermore, the status of biofilms treated with PBS or HMAPH was also investigated by fluorescent staining. Biofilms from different treatment groups were fixed with 4% glutaraldehyde overnight at 4°C, followed by removing the supernatant, rinsing with PBS, and staining with SYBR Green I for 30 min in the dark. Finally, the biofilm morphology of each treatment group was collected using a confocal laser scanning microscope (Leica TCS SP8).

**1.12. RT-qPCR analysis**

Briefly, *P. aeruginosa* (OD = 0.6-0.8) was treated separately with PBS and HMAPH (50.0 μg/mL) in the presence or absence of dual light laser irradiation for 10 min, followed by incubation for 24 h at 37 ℃. The total RNA of each treatment group was extracted using the TriQuick Total RNA Extraction Reagent (TriQuick Reagent), and the expression levels of quorum sensing (QS) system genes were quantified by RT-qPCR. The gene expression level was expressed as 2^-∆∆Ct^ and 16S was used as an internal reference. The primer sequences used in this study are shown in Table S1.

**1.13. Motility assay**

*P. aeruginosa* (OD = 0.6-0.8) was treated separately with PBS, PBS+dual light, HMAPH and HMAPH+dual light. Then, 2.0 µL of bacterial suspension was inoculated on the surface of soft agar motility plates (10 g/L tryptone, 5.0 g/L yeast extract, 10 g/L NaCl, 0.3% agar) and cultured at 37 °C for 24 h. Finally, the colony diameter of each treatment group was measured to evaluate the bacterial motility.

**1.14. Determination of pyocyanin**

The determination of pyocyanin was based on the absorbance at 520 nm of pyocyanin excreted in an acidic solution [2]. Specifically, *P. aeruginosa* (OD = 0.6-0.8) was treated separately with PBS, PBS+dual light, HMAPH, and HMAPH+dual light, and then inoculated on the slanted medium for 24 h at 37 °C. Next, 3.0 mL of chloroform was added to each group, and then pyocyanin in the chloroform phase was extracted into HCl (1.0 M) with a pink to dark red color, suggesting the presence of pyocyanin. Finally, the absorbance value at OD_520 nm_ was determined to quantify the pyocyanin content, which was expressed as OD_520 nm_×17.072.

**1.15. *In vivo* modeling of wound infection**

To further investigate the antibacterial ability of HMAPH *in vivo*, a *P. aeruginosa*-infected skin model was established on female BALB/c mice (6-8 weeks). All animal experiments were approved by the Animal Care and Experiment Committee of Tianjin University of Science & Technology (20221006). Briefly, the mice back was shaved and a circular wound about 0.6 cm in diameter was excised, followed by injection of *P. aeruginosa* (10^7^ CFU/mL, 100 μL). At 24 h post infection, the wounds were randomly divided into 4 groups (n = 10): (Ⅰ) PBS, (Ⅱ) PBS+dual light, (Ⅲ) HMAPH, (Ⅳ) HMAPH+dual light. In each treatment group, 20 μL of the corresponding liquid was evenly applied to the wound and the light group was irradiated for 30 s. On day 0, 1, 3, 5 and 7 of treatment, the mice were photographed and weighed, and each group of wounds was dipped onto LB plates for colony counting. After 7 days of treatment, all mice were sacrificed and skin tissues and major organs (heart, liver, spleen, lungs, and kidneys) were harvested. Finally, the samples were fixed with 4% paraformaldehyde solution and stained with hematoxylin and eosin (H&E) for histological analysis.
