# Supplementary material for: Thermo-responsive cascade antimicrobial platform for precise biofilm removal and enhanced wound healing
Source: Burns Trauma. 2024 Sep 25;12:tkae038. doi: 10.1093/burnst/tkae038 (PMC11422504; doi:10.1093/burnst/tkae038)
Supplement: Supplementary_material_tkae038 [file supplementary_material_tkae038.zip › Table S1 Supporting_information_tkae038.docx]

**Table S1.** Primer sequences used for RT-qPCR

| Primer | Primer sequence (5’-3’) |
| --- | --- |
| 16S-F | ACTCCTACGGGAGGCAG |
| 16S-R | ATTACCGCGGCTGCTGG |
| LasR-F | CATCGTCGCAACTACCC |
| LasR-R | GCGCACCACTGCAACACT |
| LasB-F | GTTCTATCCGCTGGTGTCG |
| LasB-R | CGCTGCCCTTCTTGATG |
| PilG-F | ACGGTTTGAAAGTGATGGTG |
| PilG-R | AAATGATGTTCGGATGGGT |
| rhlR-F | CGCCACACGATTCCCTTCA |
| rhlR-R | TCCAGACCACCATTTCCGAG |
