# Supplementary material for: Thermo-responsive cascade antimicrobial platform for precise biofilm removal and enhanced wound healing
Source: Burns Trauma. 2024 Sep 25;12:tkae038. doi: 10.1093/burnst/tkae038 (PMC11422504; doi:10.1093/burnst/tkae038)
Supplement: Supplementary_material_tkae038 [file supplementary_material_tkae038.zip › Table S2 Supporting_information_tkae038.docx]

**Table S2.** The composition of different nanosystems and corresponding abbreviations.

| Composition of nanosystems | Abbreviation |
| --- | --- |
| HMPB | — |
| HMPB@MB | — |
| HMPB@MB@AuNPs | HMA |
| HMPB@MB@AuNPs@PMB | HMAP |
| HMPB@MB@AuNPs@PMB@HA | HMAPH |
