# Supplementary figures and images for: Thermo-responsive cascade antimicrobial platform for precise biofilm removal and enhanced wound healing
Source: Burns Trauma. 2024 Sep 25;12:tkae038. doi: 10.1093/burnst/tkae038 (PMC11422504; doi:10.1093/burnst/tkae038)

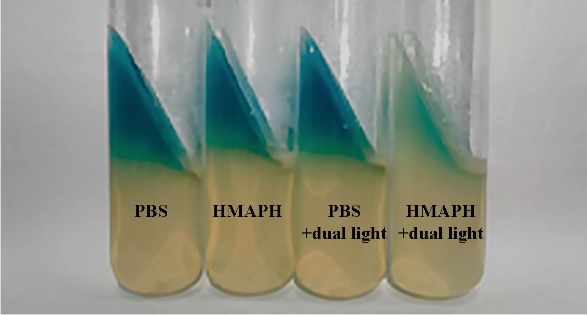


**Figure S8.** Test tube result images of *P. aeruginosa* after different treatments.

Supplement: Supplementary_material_tkae038 [file supplementary_material_tkae038.zip › Figure S8.docx]
